# Flexible Spline Models for Blinded Sample Size Reestimation in Event‐Driven Clinical Trials

**DOI:** 10.1002/pst.2459

**Published:** 2024-12-11

**Authors:** Tim Mori, Sho Komukai, Satoshi Hattori, Tim Friede

**Affiliations:** ^1^ Institute for Biometrics and Epidemiology, German Diabetes Center Leibniz Center for Diabetes Research at Heinrich Heine University Düsseldorf Germany; ^2^ German Center for Diabetes Research (DZD) München‐Neuherberg Germany; ^3^ Department of Medical Statistics University Medical Center Göttingen Göttingen Germany; ^4^ Department of Biomedical Statistics, Graduate School of Medicine Osaka University Osaka Japan; ^5^ Integrated Frontier Research for Medical Science Division, Institute for Open and Transdisciplinary Research Initiatives (OTRI) Osaka University Osaka Japan; ^6^ DZHK (German Center for Cardiovascular Research) Partner Site Lower Saxony Göttingen Germany

**Keywords:** blinded sample size reestimation, event‐driven designs, Royston–Parmar model, splines, survival extrapolation

## Abstract

In event‐driven trials, the target power under a certain treatment effect is maintained as long as the required number of events is obtained. The misspecification of the survival function in the planning phase does not result in a loss of power. However, the trial might take longer than planned if the event rate is lower than assumed. Blinded sample size reestimation (BSSR) uses non‐comparative interim data to adjust the sample size if some planning assumptions are wrong. In the setting of an event‐driven trial, the sample size may be adjusted to maintain the chances to obtain the required number of events within the planned time frame. For the purpose of BSSR, the survival function is estimated based on the interim data and often needs to be extrapolated. The current practice is to fit standard parametric models, which may however not always be suitable. Here we propose a flexible spline‐based BSSR method. Specifically, we propose to carry out the extrapolation based on the Royston–Parmar spline model. To compare the proposed procedure with parametric approaches, we carried out a simulation study. Although parametric approaches might seriously over‐ or underestimate the expected number of events, the proposed flexible approach avoided such undesirable behavior. This is also observed in an application to a secondary progressive multiple sclerosis trial. Overall, if planning assumptions are wrong this more robust flexible BSSR method could help event‐driven designs to more accurately adjust recruitment numbers and to finish on time.

## 1 Introduction

The validity of clinical trials depends on sufficiently large sample sizes to ensure a pre‐specified level of statistical power. The setting of interest here is a clinical trial with time‐to‐event outcomes, where a log‐rank test is used for the primary analysis. The sample size planning for time‐to‐event studies depends on valid estimates of the survival function [1, Ch. 15]. However, sometimes accurate information about survival rates may be unavailable at the planning stage of a trial. Changes in medical guidelines and standards of care may affect the survival rates in a given therapeutic area, which can lead to uncertainty at the planning stage of a trial.

For event‐driven designs, in which the trial finishes once a pre‐specified number of events is observed, the aim is to complete a clinical trial within a given time frame [2]. However, since the study duration is not controlled in such a design, a trial may take a significantly longer time to finish when the planning assumptions are wrong.

During the recruitment phase of a trial, a blinded sample size reestimation (BSSR) may be carried out to minimize the risk of the trial finishing late. BSSR methods use non‐comparative interim data pooled across treatment groups to adapt the design to maintain power [3]. If the event rate has been overestimated at the planning stage of an event‐driven trial, it may take longer or even be impossible to observe the required number of events within a reasonable time frame. If operationally feasible, a prolongation of the recruitment period could be considered in such a situation after carrying out a BSSR [4].

In a BSSR, the survival function across the treatment arms can be estimated from the blinded data using, for example, the Kaplan–Meier estimator. However, the estimated curve will not necessarily cover the time frame of the final analysis. Therefore, the estimated survival function needs to be extrapolated to reestimate the sample size. Some authors proposed extrapolation methods based on the Kaplan–Meier estimator, a nonparametric approach [5, 6, 7]. Although they are very flexible in estimating the survival function over the observed period of time, they are rather restrictive in terms of the extrapolation. Subsequently, parametric approaches were suggested that are less flexible in estimating the survival function but extend more naturally to the unobserved time period [2, 8, 9].

While extrapolation based on parametric models seems natural, it may not always be suitable. First, there might be a lack of external evidence to justify the choice of the parametric model. Second, patient outcomes may change compared to previous studies due to advances in medical care. Third, in some settings complex hazard functions that cannot be captured by simple parametric models occur. For example, immuno‐therapy treatments in oncology may induce delayed treatment responses and long‐term survival, which can result in complex hazard shapes [10]. In all of these cases, a flexible model that lends itself to extrapolation would be useful to make the BSSR procedure more robust.

The aim of the current study was to develop an approach for BSSR with time‐to‐event outcomes that is sufficiently flexible to capture various shapes of the survival function and can be naturally extrapolated beyond the observed time frame. Specifically, we extend the parametric BSSR framework of Friede et al. [2] by carrying out the extrapolation based on the Royston–Parmar spline model for time‐to‐event data [11]. The Royston–Parmar spline model has recently received attention in the context of survival extrapolation for health economic evaluations and has shown desirable extrapolation properties [12, 13, 14].

This article proceeds as follows: Section 2 introduces the notation and the general parametric BSSR framework. In Section 3, we propose a BSSR method based on the Royston–Parmar spline model. In Section 4, the properties of the proposed method are assessed in a simulation study in comparison to previously suggested parametric approaches. In Section 5, an application to a clinical trial in secondary progressive multiple sclerosis (SPMS) is presented. Finally, in Section 6 we discuss the implications of our findings and provide practical recommendations.

## 2 Notation and Parametric Framework

The scenario of interest is a randomized clinical trial with two treatment groups i=1,2. Let $T_{\mathrm{ij}}$ and $C_{\mathrm{ij}}$ denote the time‐to‐event and time‐to‐censoring for patient j in group i. The observation time of the patient, $t_{\mathrm{ij}}$ is the minimum of these two quantities. The event indicator, $\delta_{\mathrm{ij}}$, indicates whether the patient experienced an event ($\delta_{\mathrm{ij}}=1)$ or whether they were censored ($\delta_{\mathrm{ij}}=0$). Let $f_{i}$ and $g_{i}$ denote the density functions and $S_{i}$ and $G_{i}$ the survival functions for the time‐to‐event and time‐to‐censoring process in group i, respectively. The primary analysis is the log‐rank test and the null hypothesis is $H_{0}:\theta =1$, where θ is the hazard ratio for the treatment effect. Assuming proportional hazards, let $\theta^{*}$ denote the hazard ratio for the treatment effect under the alternative hypothesis, $H_{1}:\theta =\theta^{*}$. The required total number of events, d, to yield power 1−β can be computed as

(1) $$d=\frac{{\left(1+k\right)}^{2}}{k}\frac{{\left(z_{1-\alpha }+z_{1-\beta }\right)}^{2}}{\log\;{\left(\theta^{*}\right)}^{2}}$$

for a k:1 treatment allocation and a one‐sided significance level of α [15].

Regarding the study design, let L denote the desired end of the study, which is some time after the end of recruitment R. The origin for both quantities is the start of the trial. For example, the recruitment period may be planned to take 20 time units (e.g., days, months) and the trial may be planned to finish after 40 time units. In an event‐driven design, L is considered to be fixed, since the aim is to complete the study in the planned time frame [2]. Let $r_{\mathrm{il}}$ denote the number of patients recruited into group i per time unit l, where l=1,…,R. Note that the recruitment rate need not be uniform and the anticipated number of patients recruited per time unit may vary. Furthermore, let $D_{i}$ denote the number of events in group i at the planned end of the trial. The expected number of events in group i is given by Friede et al. [2].

(2) $$E\left(D_{i}\right)=\sum_{l=1}^{R}r_{\mathrm{il}}P\left(T_{\mathrm{ij}}<C_{\mathrm{ij}},T_{\mathrm{ij}}<L\right)=\sum_{l=1}^{R}r_{\mathrm{il}}\int_{0}^{L-l}f_{i}\left(t\right)G_{i}\left(t\right)dt$$

Assume that sometime before the end of recruitment a BSSR is carried out at a planned interim analysis. The BSSR uses the accumulated blinded data to obtain an estimate of the pooled expected number of events, $E\left(D\right)$, with $D=D_{1}+D_{2}$. This number can then be compared to the necessary number of events, d. If it is found to be too low, the planned sample size can iteratively be increased until a sufficient number of events is expected to be reached (for details on a BSSR algorithm to adapt recruitment in event‐driven designs, see Friede et al. [2].

To obtain an estimate of $E\left(D\right)$, the time‐to‐event and time‐to‐censoring processes can be modeled using an appropriate parametric function. Previously, standard parametric models such as exponential, Weibull and piece‐wise exponential models have been proposed for this purpose [2]. Since blindness needs to be maintained at the interim analysis, the group‐specific parametric functions cannot be directly estimated from the data. For the time‐to‐censoring process, we assume that it is identical across treatment groups and can thus be estimated from the pooled data. For the time‐to‐event process, previous BSSR approaches have attempted to obtain separate estimates for the two treatment groups based on a splitting procedure which relies on the assumed hazard ratio under the alternative hypothesis [2, 7].

For illustration of such a splitting procedure, consider a scenario in which there are independent exponentially distributed event times in the two treatment groups, $f_{i}\left(t\right)=\lambda_{i}e^{-\lambda_{i}t}$, where $\lambda_{i}$ is the group‐specific event rate. Based on the blinded interim data, a pooled estimate of the event rate $\bar{\lambda }$ can be obtained. Using the assumed hazard ratio under the alternative hypothesis and the known treatment allocation ratio, this pooled estimate can be split up into group‐specific estimates. Specifically, the event rate for the treatment group can be calculated as $\lambda_{2}=\frac{1+k}{1+k/\theta^{*}}\bar{\lambda }$ and the event rate of the control group can subsequently be obtained as $\lambda_{1}=\lambda_{2}/\theta^{*}$ [2].

In practice, standard parametric models (e.g., exponential models) may not be suitable and their survival extrapolations might be biased. In the next section, we propose that the flexible Royston–Parmar spline model can be used instead of standard parametric models to model the time‐to‐event and time‐to‐censoring processes. With parametric models, the survival function estimated from the blinded data is split into two treatment‐specific survival functions under the assumed treatment effect, this is neither straightforward nor necessary with the spline‐based approach. This is demonstrated in Section 3.2.

## 3 Proposed Flexible Blinded Sample Size Reestimation

### 3.1 Modeling Survival and Censoring Using the Flexible Royston–Parmar Spline Model

In this section, we consider a model for the data pooled across treatment arms for the purpose of BSSR. Define the marginal survival function as $S\left(t\right)=\frac{k}{1+k}S_{1}\left(t\right)+\frac{1}{1+k}S_{2}\left(t\right)$ and the marginal cumulative hazard function as $H\left(t\right)=-\log S\left(t\right)$. The Royston–Parmar spline model [11] is a flexible parametric model for time‐to‐event outcomes, which uses restricted cubic splines to model the log cumulative hazard function $\log\left\{H\left(t\right)\right\}$. It is a generalization of the Weibull proportional hazards model and provides a useful alternative to the commonly used semi‐parametric Cox proportional hazards model. While being more flexible than standard parametric models, it maintains the advantages of explicitly modeling the baseline hazard function, which allows for extrapolations of the survival probability beyond the last observed event time in a sample [11].

Suppose we observe some failure times in the interim data and let the log‐transformed failure times be denoted by $y=\log\left(t\right)$. The smallest and largest log‐transformed failure times are used as boundary knots for the restricted cubic spline. The flexibility of the spline model is determined by the number of internal knots placed between the boundary knots. In between neighboring knots, a cubic function is assumed and beyond the boundary knots a linear function is assumed [11]. Let $c_{\min}$ and $c_{\max}$ denote the boundary knots and let $c_{1}<\ldots <c_{m}$ denote the internal knots such that m is the number of internal knots. Corresponding to the number of internal knots, the spline model contains m non‐linear terms $\nu_{j}\left(y\right)$, where j=1,2,…,m [11].

The Royston–Parmar spline model [11] is defined as

(3) $$\log\left\{H\left(t\right)\right\}=\gamma_{0}+\gamma_{1}y+\gamma_{2}\nu_{1}\left(y\right)+\ldots +\gamma_{m+1}\nu_{m}\left(y\right)$$

where the *j*th non‐linear term is defined as

$$\nu_{j}\left(y\right)={\left(y-c_{j}\right)}_{+}^{3}-\psi_{j}{\left(y-c_{\min}\right)}_{+}^{3}-\left(1-\psi_{j}\right){\left(y-c_{\max}\right)}_{+}^{3}$$

with

$${\left(y-a\right)}_{+}=\max\left\{0,\left(y-a\right)\right\}$$

for any value a, and

$$\psi_{j}=\frac{c_{\max}-c_{j}}{c_{\max}-c_{\min}}$$

The m+2 unknown parameters $\gamma_{0},\gamma_{1},\ldots ,\gamma_{m+1}$ can be estimated using standard maximum likelihood methods [11]. The internal knots are spaced equally between percentiles of the distribution of the log‐transformed failure times [11]. For example, in a 2‐knot model, the knots would be placed at the 33% and 67% percentiles and in a 3‐knot model at the 25%, 50%, and 75% percentiles of the log‐transformed failure times. Royston and Parmar [11] recommend one internal knot as a reasonable initial modeling choice. They point out that often a significant improvement over a simple Weibull model is obtained by adding one knot, but adding further knots does not always further improve the model fit [11]. In practice, it has been suggested that 1–4 internal knots should be used depending on the desired model flexibility [16]. Note that when a 0‐knot model is fitted, the model reduces to a linear function of the log cumulative hazard function in log time and thus a Weibull function [11].

Regarding extrapolation, the restricted cubic splines imply that the values beyond the boundary knot $c_{\max}$ follow a local Weibull distribution. The linear trend in the restricted cubic splines arises as a continuation of the last cubic polynomial given the continuity restrictions imposed by the model. In practice, this may lead to more realistic survival and censoring extrapolations compared to extrapolations based on standard parametric models.

### 3.2 Blinded Sample Size Reestimation (BSSR) Based on a Single Spline Model Fit to the Pooled Data

As a further modification to existing BSSR approaches [2, 7], we propose that the total expected number of events $E\left(D\right)=E\left(D_{1}\right)+E\left(D_{2}\right)$ can be estimated directly by using the pooled estimate of the survival function from the blinded data. That is, the BSSR can be carried out without the need for a procedure to split the estimated survival function into group‐specific estimates. To see why this step can be skipped in a BSSR algorithm consider the following. Let $r_{l}$ denote the recruitment numbers per time unit l pooled across both treatment arms. At the time point of the interim analysis, the recruitment numbers are known for the recruitment time units observed so far. Recruitment in future time units must be projected based on available information and suitable models (for a review of recruitment prediction models, see Gkioni et al. [17]). Then, based on the known treatment allocation ratio the group‐specific recruitment numbers per time unit can be approximated as $r_{1l}\approx r_{l}\frac{k}{k+1}$ and $r_{2l}\approx r_{l}\frac{1}{k+1}$. Using this approximation, the total expected number of events can be obtained as
(4)

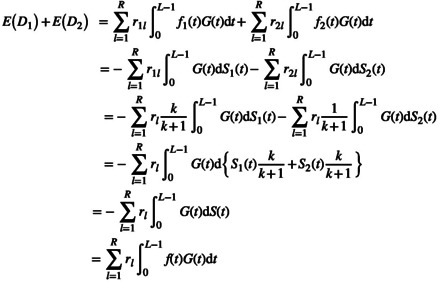




**FIGURE 1:**
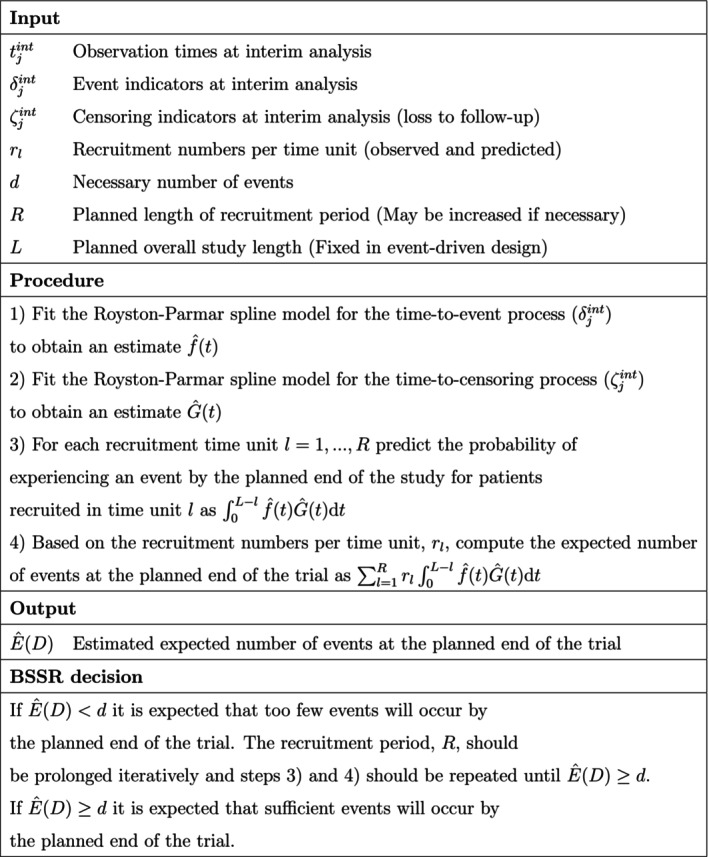
Algorithmic overview of the flexible BSSR method. Example code and example data (interim data from our case study, see Section 5) are available in the Supporting information.

Thus, to estimate the total number of events the pooled estimate of the survival function from the blinded data, $S\left(t\right)$, can be used directly and a splitting procedure is not necessary. Therefore, the model that is fit to the pooled survival data during an interim analysis can be used directly to extrapolate the survival times, which offers a simplification compared to existing BSSR approaches. For illustration, an algorithmic overview of the proposed flexible BSSR method is depicted in Figure 1. Note that a splitting procedure as previously proposed would complicate the procedure by requiring an extra step after estimating $f\left(t\right)$ and subsequently requiring steps (3) and (4) to be carried out separately for the two treatment groups.

When a Royston–Parmar spline model is used to model the time‐to‐event and time‐to‐censoring process, an analytic solution to the integral $\int_{0}^{L-l}\hat{f}\left(t\right)\hat{G}\left(t\right)dt$ is not available. Therefore, numerical integration is used to obtain the expected number of events based on the proposed flexible BSSR method.

## 4 Simulation Study

We carried out a simulation study to investigate the operating characteristics of our proposed flexible BSSR method in comparison with standard parametric BSSR as well as a fixed sample size design [18]. The general simulation setup was based on the simulation study of Friede et al. [2], who designed it to mirror the event‐driven EXPAND trial for SPMS [19]. We extended the simulation setting to investigate the performance of standard parametric BSSR and flexible BSSR under different data‐generating mechanisms with non‐constant hazards. Detailed information regarding the assumptions for data generation, design options, and the software used are available in Supporting Information: Appendix A.1.

### 4.1 Set Up of the Simulation Study

#### 4.1.1 Assumptions for the Data Generation

We generated data under three different scenarios: (1) Exponential survival, (2) Weibull survival, or (3) Gompertz survival. The censoring times were generated from an independent exponential distribution in all three scenarios. In line with the motivating SPMS study, we assumed that the timeline of the trial was defined in terms of months. The event rates of the control group were varied such that the probability of experiencing an event by 24 months was between 20% and 30%. The probability of experiencing censoring by 24 months was fixed to be 20% for both treatment groups. In the Weibull and Gompertz simulation scenarios the event percentages of 20%, 21%, …, 30% were generated by varying the shape parameter of the respective distribution. For the Weibull and Gompertz scenarios, we simulated data with increasing and decreasing hazards. The parameter sets for the data generation in the different simulation scenarios are available in Supporting Information: Appendix A.2. Graphs of the survival and hazard functions of the models used for data generation are available in Supporting Information: Appendices A.4–A.6.

The initial recruitment plan of the 1530 patients is based on the planning assumption that the percentage of events at 24 months in the control group is 30%. When these planning assumptions hold, 374 events are expected to occur within the planned 39 months of follow‐up. When the event probability is < 30%, however, the trial is expected to take longer than 39 months and additional recruitment is necessary.

#### 4.1.2 Design Options

A blinded review was carried out at Month 18, shortly before the planned end of the recruitment period at Month 20. At that point, 1320 of the planned 1530 patients were already recruited into the study. We compared the following designs: (1) flexible BSSR, (2) Weibull BSSR, (3) exponential BSSR, and (4) fixed sample size design. For the flexible BSSR, the Royston–Parmar spline model for the time‐to‐event process was fit with 1, 2, and 3 internal knots. The Royston–Parmar spline model for the time‐to‐censoring process was fit with 1 internal knot.

#### 4.1.3 Metrics

First of all, we assessed the mean relative bias in the estimated number of events for each of the BSSR methods. The relative bias in each simulation run was calculated as

(5) $$\frac{\hat{E}\left(D\right)-E\left(D\right)}{E\left(D\right)}$$

$E\left(D\right)$ was calculated with Equation (2) based on the true group‐specific parameter values used for the data generation. $\hat{E}\left(D\right)$ is the estimate of the expected number of events based on the parameter estimates of a given model (e.g., estimated exponential event rate in the exponential BSSR method). For the exponential and Weibull BSSR methods, it was also calculated based on Equation (2) using a splitting procedure. For the flexible BSSR method, the pooled estimate of the survival function was directly used without a splitting procedure as proposed in Equation (4). As additional metrics, we compared the mean trial duration and the mean number of additional patients recruited based on the BSSR procedure. Moreover, we assessed the rejection probability, which was calculated as the proportion of simulated trials in which the log‐rank test rejected the null hypothesis. Depending on whether the data were generated under the alternative or the null hypothesis, this corresponded to the power or the type I error rate, respectively.

### 4.2 Simulation Results

In the following sections, we will focus on the results of the Weibull and Gompertz simulations, since exponential BSSR simulations have been considered in previous simulation studies. We present the performance of the standard parametric BSSR and flexible BSSR in settings with decreasing and increasing hazards. When data were generated under the null hypothesis $\left(\theta =1\right)$, we found no inflation of the type I error rates for the BSSR designs. The rejection probability was close to the nominal significance level of 0.05 in all simulation scenarios (10,000 replications per scenario under the null hypothesis, see Supporting Information: Appendix A.3). The sections below report the results of the simulations in which the data were generated under the alternative hypothesis $\left(\theta =0.7\right)$. A sensitivity analysis to investigate the effect of the timing of the interim analysis (earlier interim analyses with less data available) is presented at the end of the Gompertz simulation results section.

#### 4.2.1 Weibull Simulation

As anticipated, the exponential BSSR method was biased in the Weibull simulation scenarios. It over‐ and underestimated the estimated number of events at trial end in the decreasing and increasing hazard scenarios, respectively. The bias became larger the stronger the constant hazards assumptions were violated. In terms of additional recruitment at the interim analysis, the underestimation of the estimated number of events led the exponential BSSR to add unnecessarily many patients. While some unnecessary additional recruitment may be expected due to sampling variability, this was much more pronounced for the biased exponential BSSR. In the scenario with the most strongly increasing hazards (30% events, Weibull shape parameter of 1.148), the exponential model added on average 429 Patients, whereas all other BSSR models added less than 250 patients on average. By contrast, the overestimation of events by the exponential BSSR in the decreasing hazards scenarios was less problematic. On average, it took the exponential BSSR only about 1 month longer to finish than the (unbiased) Weibull BSSR. The moderately flexible 1‐knot spline BSSR performed similarly to the Weibull BSSR, whereas the more flexible 2–3 knot spline models performed slightly worse due to occasional overfitting. More detailed results and graphs for the Weibull simulations can be found in Supporting Information: Appendix A.5.^1^

#### 4.2.2 Gompertz Simulation

##### 4.2.2.1 Gompertz Decreasing Hazards

**FIGURE 2:**
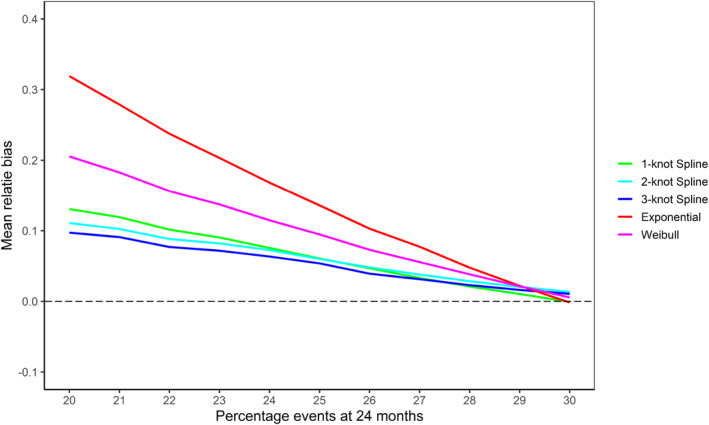
Mean relative bias in the estimated number of events at trial end in the simulated decreasing hazards Gompertz scenarios based on the BSSR models (10,000 replications per scenario). The Royston–Parmar spline model was fit with 1, 2, and 3 knots. Note that no sample size reestimation was carried out in the fixed design. The expected number of events in the simulation scenarios ranged from 239.1 (20% events) to 372.3 (30% events).

The mean relative bias in the estimated number of events at trial end in the decreasing hazards Gompertz scenarios is shown in Figure 2. As the shape parameter of the Gompertz distribution decreases, all BSSR methods start to overestimate the number of events. This effect is the most pronounced for the exponential model, which overestimates the number of events by 31.9% in the most extreme simulation setting (20% events, Gompertz shape parameter of −0.0427). The Weibull model overestimated the number of events by a somewhat smaller amount of 20.5% in the same scenario. The spline models had the lowest bias and overestimated events by between 9.1% and 11.9% in that scenario.

**FIGURE 3:**
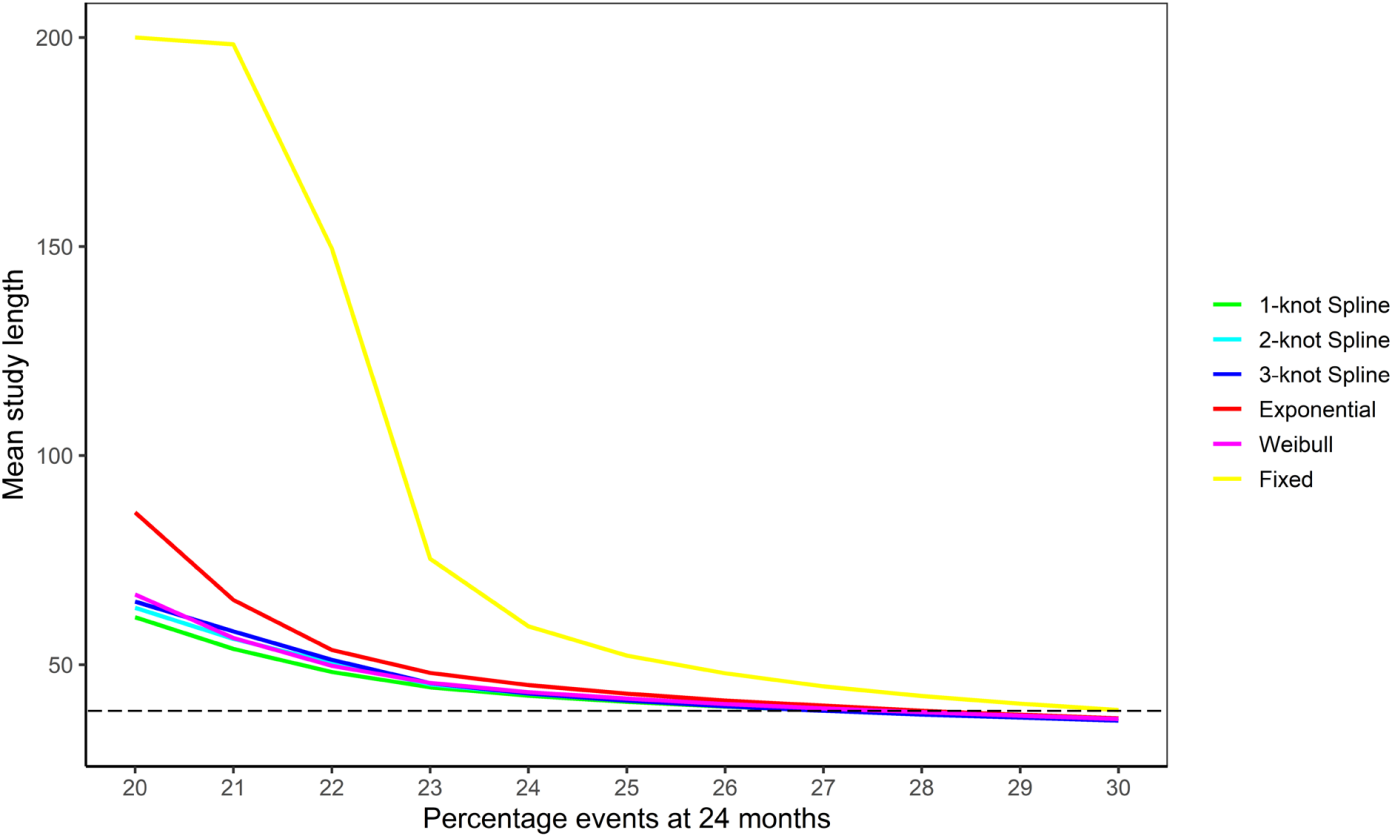
Mean study lengths in the Gompertz scenarios with decreasing hazards based on the BSSR models and the fixed design (10,000 replications per scenario). The trial finished once 374 events were observed or after a maximum trial duration of 200 months (only relevant for the decreasing hazards Gompertz scenarios). The goal was to finish in 39 months (black dotted line).

As for the mean trial length (Figure 3), the fixed design took an extremely long time when the Gompertz shape parameter became small. In the 20% event scenario, the fixed design (without a BSSR) always ran until the maximum trial duration of 200 months. That is, the necessary number of 374 events was never observed within a trial duration of 200 months and the final analysis had to be carried out with insufficient events. While all BSSR methods performed better than that, the exponential BSSR still took an average 86.4 months in the 20% events scenario. The Weibull BSSR took on average 66.8 months and the spline models took 61.3 months (1‐knot Spline), 63.6 months (2‐knot Spline), and 65.1 months (3‐knot Spline). Therefore, the 1‐knot spline model performed best in terms of average trial duration.

##### 4.2.2.2 Gompertz Increasing Hazards

**FIGURE 4:**
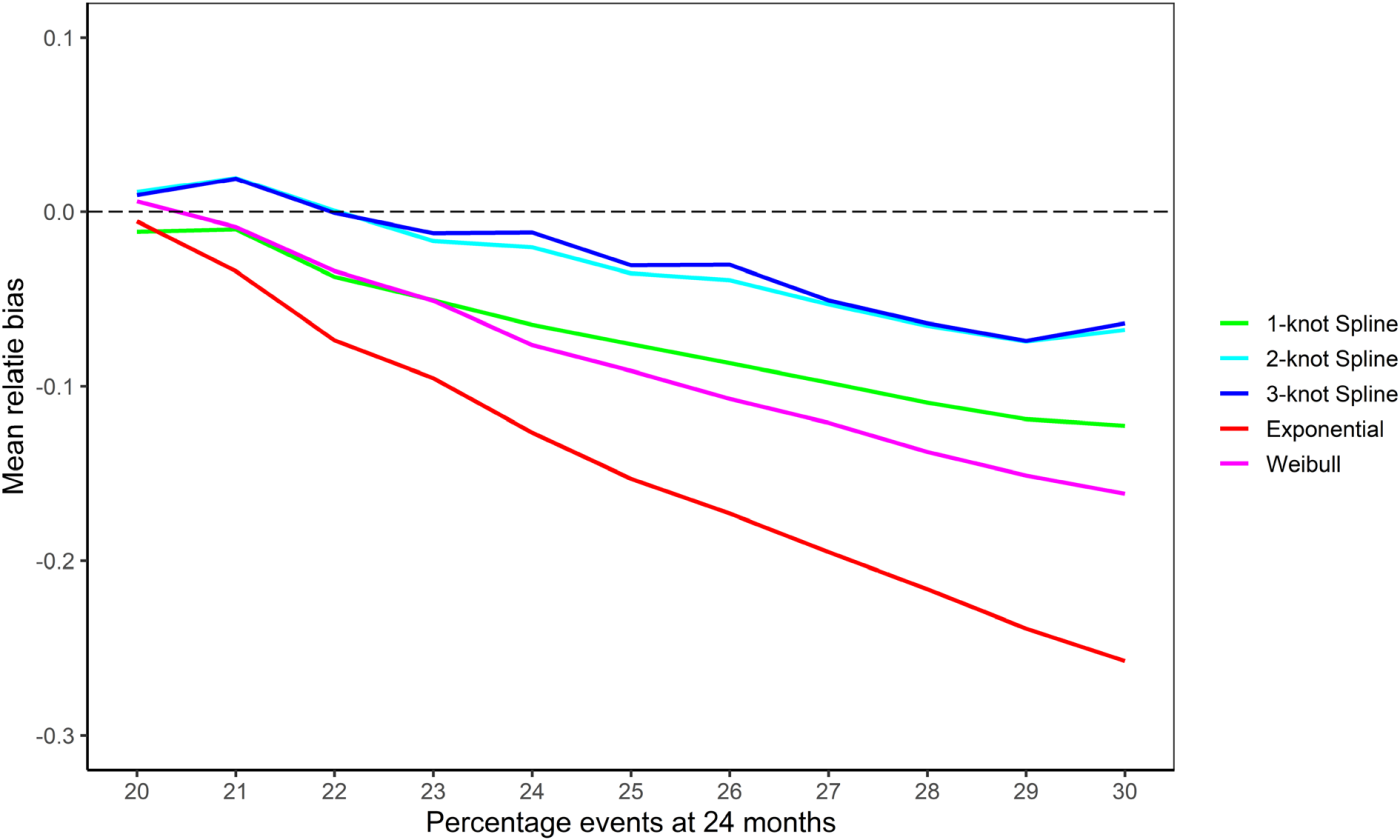
Mean relative bias in the estimated number of events at trial end in the simulated increasing hazards Gompertz scenarios based on the BSSR models (10,000 replications per scenario). The Royston–Parmar spline model was fit with 1, 2, and 3 knots. Note that no sample size reestimation was carried out in the fixed design. The expected number of events in the simulation scenarios ranged from 246.8 (20% events) to 389.9 (30% events).

The mean relative bias in the estimated number of events at trial end in the increasing hazards Gompertz scenarios is shown in Figure 4. The increasing Gompertz shape parameter results in hazards that increase strongly over time and all BSSR methods underestimated the number of events in these simulation scenarios. The exponential BSSR again had the largest bias, underestimating the number of events at the trial end by 25.6% in the scenario with the largest Gompertz shape parameter (30% events, Gompertz shape parameter of 0.0364). The average bias of the Weibull BSSR was considerably lower at 13.7%. The 1‐knot spline model had a slightly lower average bias of 10.9% and the 2‐ and 3‐knot spline models had the lowest average bias of 6.5% and 6.3%, respectively.

**FIGURE 5:**
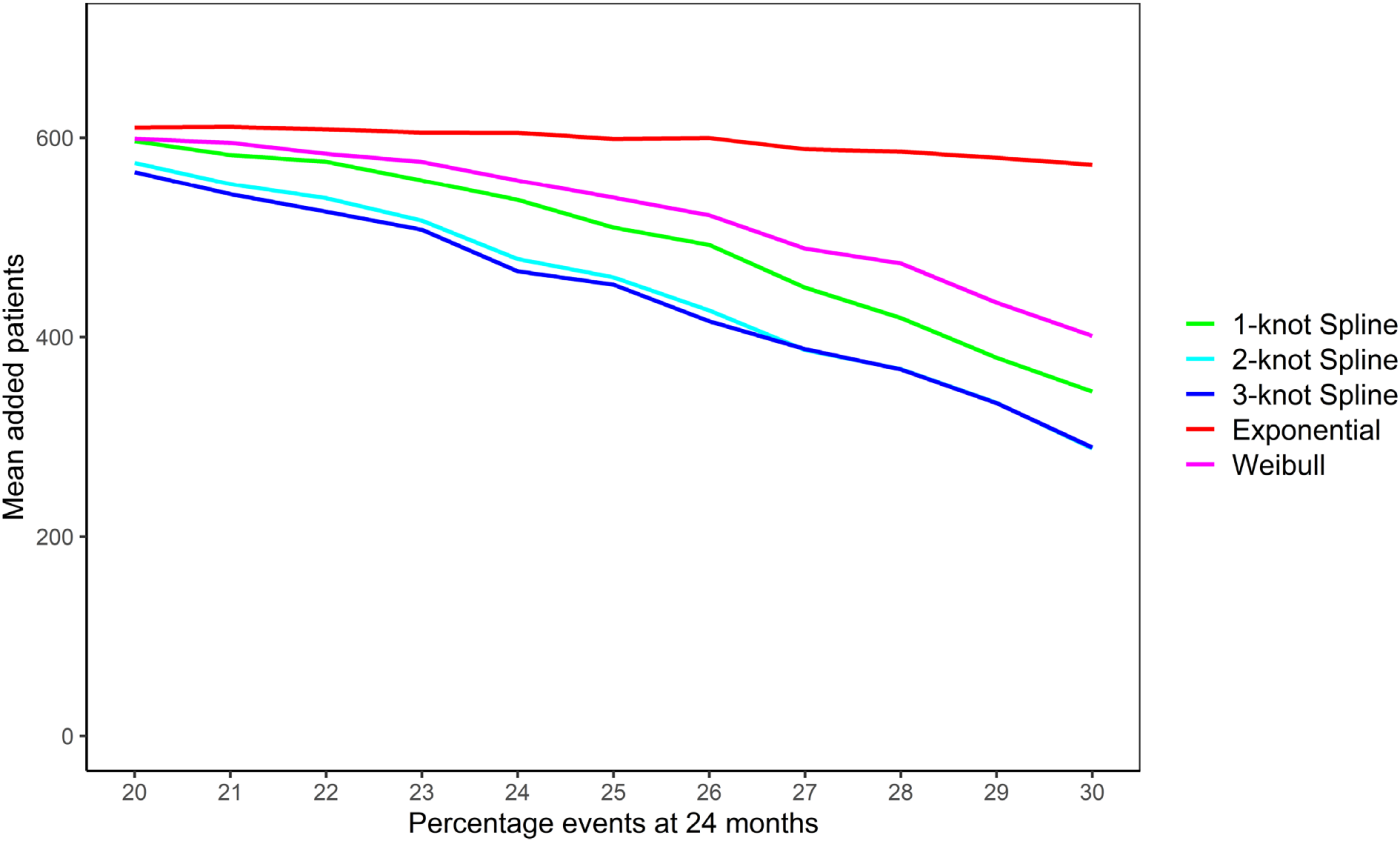
Mean number of additional patients in the Gompertz scenarios with increasing hazards added by the different BSSR models (10,000 replications per scenario). The maximum number of patients that could be added was 612. Note that no patients were added in the fixed design.

The different degrees of underestimation are reflected in the average number of patients added by the different BSSR methods (Figure 5). The exponential BSSR almost always added about 600 patients on average, even when the true expected number of events was close to the anticipated 30% and additional recruitment was not necessary. The average number of additional patients for the remaining models decreases somewhat as the percentage of events in the data increases. In the most extreme increasing hazards Gompertz scenario the 2‐ and 3‐knot spline models added on average 288 and 299 patients. The 1‐knot spline model added on average 345 patients and the Weibull model 401 patients. Therefore, while all BSSR methods recruited unnecessary additional patients in this scenario, this was less pronounced for the flexible BSSR methods, which were better able to capture the increasing hazard function. More detailed results and graphs for the Gompertz simulations can be found in Supporting Information: Appendix A.6.

##### 4.2.2.3 Earlier Interim Analyses With Smaller Sample Sizes

Given the relatively large sample size available at the interim analysis of our simulated trial (1320 patients at 18 months), we carried out a sensitivity analysis for the Gompertz simulation study by performing the interim analysis at earlier time points (16, 14, and 10 months). The resulting sample sizes available at the interim analyses were 1110, 903, and 495 patients, respectively. When fewer data were available for model estimation, we observed that the flexible spline model could sometimes not be estimated (Supporting Information: Appendix A.6, Figure A.18). While this seemed less prominent when the interim analysis was carried out after 16 or 14 months (estimation failed in max. 3% of iterations), the issue was aggravated if an early interim analysis was carried out after 10 months of recruitment. In the simulation setting with the fewest events (20% events at 24 months, interim analysis after 10 months) the flexible spline model could not be estimated in 12.5% of the simulation runs. It is likely that in simulation runs with fewer events available at the interim analysis there were numerical problems with estimating the flexible spline model with up to 3 internal knots.

## 5 Case Study in SPMS

To illustrate our proposed flexible BSSR method, we applied it to the data of the SPMS trial, which have been published by Kappos et al. [19]. Multiple sclerosis (MS) is a chronic neurological condition [19]. SPMS is a later stage of the disease, which is associated with a continuous progression of physical disability and neurological deficits [19]. The study by Kappos et al. [19] was a large phase 3, randomized, double‐blind, and placebo‐controlled trial. The design was event‐driven and the time‐to‐event analysis was planned for when a minimum of 374 events had been observed [19]. The primary endpoint was 3‐month confirmed disability progression (CDP), which was quantified as a relevant increase in the Expanded Disability Status Scale (EDSS) score [19]. A 2:1 randomization (verum:control) was used for treatment assignment. At the time‐to‐event analysis, 1096 patients had been assigned to the treatment group and 545 patients to the control group [19].

Since individual patient data (IPD) were not available to us, we obtained pseudo‐IPD based on the published Kaplan–Meier curves using an extraction algorithm [20]. The algorithm uses digitized Kaplan–Meier curves in combination with available information on numbers of events and numbers at risk at given intervals. The pseudo‐IPD are obtained numerically by solving the inverted Kaplan–Meier equations [20]. The digitized data for the two treatment arms from the Kaplan–Meier plot were obtained by using the WebPlotDigitizer app [21]. We used the cumulative enrollment plot provided in the appendix of Kappos et al. [19] as a basis to generate similar enrollment times using piece‐wise uniform distributions (Supporting Information: Appendix A.7, Figure A.19). The generated enrollment times were randomly assigned to the pseudo‐IPD.

For our BSSR application, we assumed that a BSSR would take place after 18 months of recruitment, which would be 2 months prior to the planned end of recruitment. The corresponding interim analysis dataset thus only included patients and their information up until that recruitment month. The full dataset was used to evaluate how well the BSSR method projected into the future. If the respective parametric models indicated that fewer than 374 events were expected at 39 months, the corresponding BSSR considered additional recruitment of up to 6 months. For the BSSR methods we again considered standard parametric BSSR (exponential and Weibull) and flexible BSSR. For the flexible BSSR, the Royston–Parmar spline model for the time‐to‐event process was fit with 1–3 internal knots and the model for the time‐to‐censoring process was always fit with 1 internal knot. Model fit to the interim data was assessed using the Akaike Information Criterion (AIC) and Bayesian Information Criterion (BIC). The AIC is defined as $\mathrm{AIC}=-2\log\hat{L}+2q$, where $\hat{L}$ denotes the likelihood and q denotes the number of parameters of the model [22]. The BIC is defined as $\mathrm{BIC}=-2\log\hat{L}+\log\left(n\right)q$, where n denotes the number of uncensored observations [1, Ch. 3].

**FIGURE 6:**
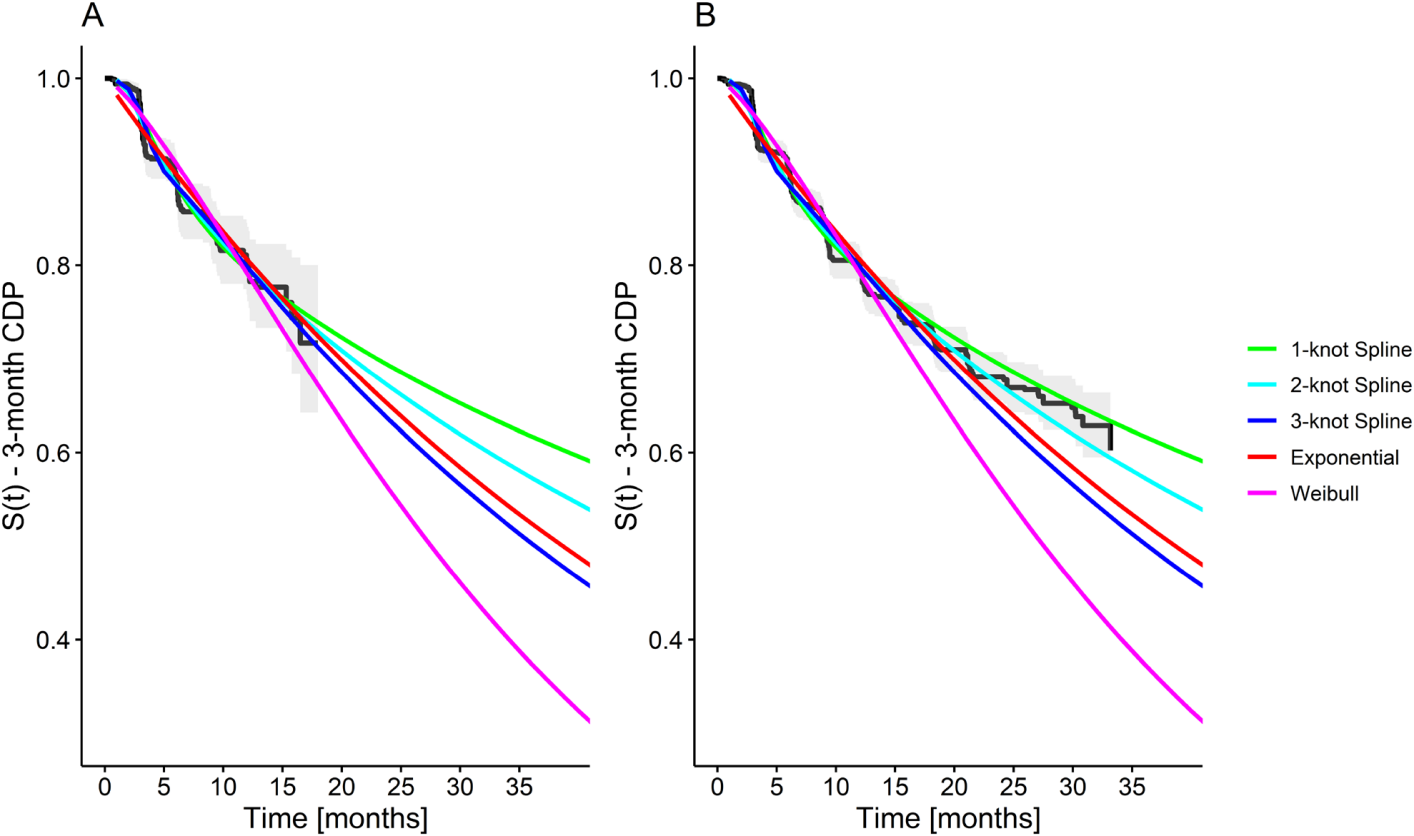
Survival modeling of the interim data of the secondary progressive multiple sclerosis (SPMS) data based on standard parametric models (Exponential and Weibull) as well as the Royston–Parmar spline model with 1, 2, or 3 knots. The predicted values based on the different models are shown in comparison to the Kaplan–Meier curve of (A) the interim data, and (B) the full dataset.

Figure 6 shows the fits of the different parametric models for the survival distribution in comparison to the Kaplan–Meier estimates based on the interim dataset and the full dataset.

While the predicted values for the survival function based on the different parametric models (Figure 6A) appear relatively similar for the first 18 months, the extrapolations of survival at the planned trial end at 39 months differ by a large margin. For example, the 1‐knot spline model predicts a survival probability of 60% at 39 months, whereas the Weibull model predicts a survival probability of only 34%.

When comparing the Kaplan–Meier curve of the full dataset to the model‐based extrapolations (Figure 6B), we can see that survival at the last observed event, $S_{\mathrm{KM}}=0.60$, was underestimated by several models. There was some underestimation by the exponential $\left(S_{\text{Exponential}}=0.55\right)$ and 3‐knot spline model $\left(S_{3-\text{knotSpline}}=0.53\right)$, but a much more extreme underestimation by the Weibull model $\left(S_{\text{Weibull}}=0.41\right)$. In contrast, the 2‐knot spline model $\left(S_{2-\text{knotSpline}}=0.59\right)$ had a very close estimate and the 1‐knot spline model $\left(S_{1-\text{knotSpline}}=0.63\right)$ slightly overestimated survival.

An inspection of the estimated hazard functions (Supporting Information: Appendix A.7, Figure A.20) showed that the Weibull model estimated hazards that increased over time, with a Weibull shape parameter of 1.31. In contrast to this, the spline models estimated an initial sharp increase followed by a sharp drop in the hazard rate. This suggests that the moderately flexible Weibull model, which is restricted to monotonous hazard functions, was not able to capture the hazard rate shape observed in the data. The simple exponential model estimated a constant, relatively low hazard rate, which was similar to the lower hazard predicted by spline models for later part of the follow‐up.

Table 1 shows the AIC and BIC values for the different parametric models for the survival function of the SPMS data. The 1‐knot spline model had the lowest BIC and the 3‐knot spline model had the lowest AIC. These conflicting recommendations of the AIC and BIC highlight a practical challenge when selecting a model for survival extrapolation. Good within‐sample fit does not necessarily guarantee a good extrapolation, as illustrated by the relatively poor extrapolation of the 3‐knot spline model in this example. This may be due to overfitting of local trends in the data, which a spline model may become increasingly prone to as the number of internal knots increases. Therefore, a more conservative information criterion like the BIC might be a better choice to try and prevent overfitting. We return to a discussion of model selection and model flexibility in the discussion section.

**TABLE 1:** AIC and BIC values for the different parametric models for the survival function of the SPMS data.

Model	AIC	BIC
1‐Knot Spline	996.2	1012.4
2‐Knot Spline	997.2	1018.9
3‐Knot Spline	987.3	1014.3
Exponential model	1026.5	1031.9
Weibull model	1018.6	1029.4

*Note*: The 3‐knot Spline model had the lowest (best) AIC and the 1‐knot Spline model had the lowest (best) BIC value.

Based on the model estimates of S(t) and G(t) (Supporting Information: Appendix A.7, Figure A.21) we estimated the expected number of events at the planned trial end at 39 months for each of the five models and ran the BSSR algorithm. To evaluate the BSSR decisions we constructed hypothetical datasets in which additional patients from the full dataset were added if the recruitment period was prolonged. Moreover, we assessed when the trial would have finished if the BSSR had been carried out based on the respective model. The results are summarized in Table 2.

**TABLE 2:** Expected number of events estimated by the different BSSR models and resulting trial characteristics.

BSSR model	Ê (D)	${\hat{\sigma }}_{\hat{E}\left(D\right)}$	R	*N*_Add_	*L*_Obs_	$D_{t=39}$
Exponential	299.0	24.9	26	288	35.3	401
Weibull	372.0	34.5	21	48	NA	314
1‐Knot Spline	251.7	30.2	26	288	35.3	401
2‐Knot Spline	266.3	36.6	26	288	35.3	401
3‐Knot Spline	290.6	38.4	26	288	35.3	401

*Note*: ${\hat{\sigma }}_{\hat{E}\left(D\right)}$ denotes the bootstrap standard error of the E(D) estimates based on 1000 bootstrap samples. R denotes the length of the recruitment period and *N*_Add_ denotes the number of additional patients that were recruited due to the BSSR decision. *L*_Obs_ is the observed trial length (the time at which 374 events would have been observed) in the hypothetical dataset if the enrollment period had been extended based on the respective BSSR model. $D_{t=39}$ is the number of events that would have been observed after 39 months.

The expected number of events at the planned end of study at 39 months was estimated to be less than the necessary 374 events by all models. However, the estimated expected number of events varied considerably from 251.7 events (1‐knot spline model) to 372 events (Weibull model). While the estimate of the Weibull model was close to the necessary 374 events, the estimates of all other models were considerably lower with a maximum of 299 events (exponential model). The BSSR algorithm based on the exponential model and all three spline models indicated that the enrollment period should be increased by the maximum of 6 months (288 additional patients). In contrast, the BSSR algorithm based on the Weibull model only resulted in the enrollment period being prolonged by 1 month (48 additional patients). Given their identical BSSR decisions, the other models had identical trial characteristics with the trial finishing well on time after 35.3 months. In contrast, the Weibull BSSR, with only minor additional recruitment, did not reach 374 events at all and only recorded 314 events after 39 months due to censoring.

The bootstrap standard error of the model estimates of the expected number of events ranged from 24.9 (exponential model) to 38.4 (3‐knot spline model). This illustrates that the increased flexibility of the models comes at the cost of increased variability. However, for the 1‐knot spline model we only find a moderately increased standard error compared to the simple exponential model. Of note, the standard error of the 1‐knot spline model (30.2) was smaller than that of the Weibull model (34.5), since the latter was inflated due to the strong overestimation of the expected number of events. This further supports the use of a moderately flexible 1‐knot spline model as it strikes a good balance between flexibility and stability. Overall, this case study shows that the Royston–Parmar spline model performs well in the context of BSSR in this real‐world data setting. In particular, the flexible modeling approach may be useful when there are reasons to believe that the underlying hazard function is non‐monotonous.

## 6 Conclusions and Discussion

The aim of the current article was to propose a flexible approach for BSSR in clinical trials with time‐to‐event outcomes and to compare it to existing parametric approaches. Here we proposed an extension of Friede et al.'s [2] parametric BSSR framework by carrying out the extrapolation based on the Royston–Parmar spline model.

To investigate the operating characteristics of our proposed method under different data‐generating mechanisms, we carried out a simulation study motivated by a large phase 3, randomized clinical trial for the treatment of SPMS [19]. Here, we observed that using a simple exponential model for the BSSR could lead to problems when the constant hazards assumption was violated (e.g., when the survival function followed a Weibull or Gompertz distribution). In the interim analysis setting considered here (1320 out of 1530 patients recruited over a period of 18 months), we found that a moderately flexible 1‐knot spline model performed well across the simulations as it struck a good balance between flexibility and stability. When we simulated data under the null hypothesis (10,000 replications per scenario), we found no indication of an inflation of the type I error rate, which is in line with previous simulation studies of BSSR for clinical trials with time‐to‐event outcomes [2, 5, 8]. Concerning the use of pooled parameter estimates from the blinded data for the purpose of BSSR, we found that our proposed flexible parametric BSSR performed well without any splitting procedure. This strengthens our argument that a splitting procedure as proposed by Friede et al. [2] and Whitehead et al. [7] is not necessary when carrying out a BSSR.

While our simulated study included a variety of survival distributions with different shapes for the hazard function, it is important to acknowledge that one specific trial setting was used as the basis for the simulation study. While our proposed flexible BSSR method performed well in this setting of a large clinical trial (planned recruitment of 1530 patients and 374 events expected at the end of follow‐up), the generalizability of these findings warrants further discussion. For example, when we simulated a BSSR at an earlier time point (10 months) for the Gompertz simulation, the flexible spline models could not be estimated in up to 12.5% of the simulation runs, as fewer events were present in the data. To expand on the issue of generalizability, we reviewed other studies that examined the Royston–Parmar spline model and its extrapolation performance across settings with various sample sizes and follow‐up periods. We identified four simulation and real‐world studies, which are summarized in Table A9 (Supporting Information: Appendix A.8). Note that these simulation studies were carried out from an economic evaluation perspective and therefore considered longer extrapolation periods than are common in a BSSR setting. Overall, these studies highlight that a flexible spline model will not necessarily outperform standard parametric models, even when the simulated data follow a complex hazard shape [14, 23]. While the spline model seemed relatively robust to both smaller sample sizes and shorter follow‐ups, the risk of overfitting increases if increasingly flexible spline models (> 1 knot) are used in settings where less data is available [14, 23]. Moreover, there might be less or even no gain compared to standard parametric models when fewer information is available to estimate the hazard function [24]. This suggests that the proposed spline‐based BSSR method might be relevant primarily in settings in which (1) a decent sample size and follow‐up data are available and (2) there are reasons to assume that standard parametric models are not well suited for survival extrapolation.

A further limitation of our study is that we only compared the proposed flexible BSSR method to BSSR methods based on standard parametric models, which were previously proposed in the parametric framework of Friede et al. [2]. However, non‐parametric BSSR methods (see e.g., Whitehead et al. [7] and Todd et al. [5]) provide an alternative, robust method for BSSR in time‐to‐event settings. Therefore, a more comprehensive simulation study, which considers a broader set of clinical trials and includes non‐parametric BSSR methods as a comparator, would be an important avenue of future research.

An important point to consider when planning a BSSR for a clinical trial is the time point at which the interim analysis should be carried out. In principle, it would be optimal to have as much follow‐up information as possible to estimate and extrapolate the survival function. The less follow‐up information is available the less reliable the estimate of the survival function and thus the re‐estimated sample size will be [25]. On the other hand, from a logistical standpoint it is undesirable to complete a sample size review too late in a study, potentially after the end of the initially planned enrollment period [26, 27]. Given the data preparation and processing time necessary for a BSSR, Pritchett et al. [27] recommend that an interim analysis should be scheduled at least 2 months before the projected end of enrollment. Moreover, it is recommended to perform trial simulations to assess the design options such as the timing of the interim analysis across a variety of settings [27]. Specific recommendations for trial simulations for BSSR designs are available in Mayer et al. [28].

We note that a similar spline‐based modeling approach for time‐to‐event outcomes to that of Royston and Parmar [11] has been proposed by Crowther and Lambert [29]. However, for the purpose of extrapolating survival we deemed the Royston–Parmar spline model more suitable, since it relies on modeling the log cumulative hazard function rather than the less stable log hazard function [11]. While the generalization of the Weibull model presented here is the most commonly used parametric spline model for time‐to‐event data, Royston and Parmar [11] also introduced a spline‐based generalization of the log‐logistic model. While the former model is a flexible proportional hazards model (PH spline model), the latter is a flexible proportional odds model (PO spline model) [11]. The PO spline model provides an alternative extrapolation mechanism, since the part beyond the right boundary knot follows a local log‐logistic distribution [11].

To conclude, we would like to provide some practical recommendations for using the Royston–Parmar spline model for BSSR in event‐driven designs. The BIC can be used as a guidance to determine the flexibility of the spline model, but it should not be exclusively relied on. It is important to bear in mind that a good within‐sample fit does not guarantee a good extrapolation performance [14, 23, 24]. A 1‐knot spline model can be a useful starting point if one is uncertain about the validity of the exponential or Weibull model for the data at hand [11]. Very flexible spline models (2–3 internal knots) should be used with caution and only if there is reason to assume complex hazard shapes, because overfitting is a concern with such flexible models. This is likely to be more problematic in smaller samples with fewer events, where sampling variability is larger. If the hazard function hardly changes with a higher number of internal knots, this is a sign of overfitting and a simple model should be preferred [16]. In practice, fitting the spline models on different scales (PH scale and PO scale) should be considered [13]. In general, the model should not only be chosen in terms of optimal within‐sample fit, but clinical or biological plausibility as well as external evidence should be taken into account [30].

## Conflicts of Interest

T. F. provided consultancies to Novartis Pharma AG regarding sample size reestimation strategies for the MS study that served as an example in this article. Otherwise, the authors declare no conflicts of interest.

## Supporting information


**Data S1** Supporting Information.

**Data D2** Supporting Information.

**Data D3** Supporting Information.

## Data Availability

The data of the example study are publicly available and the generated interim data are available in the online Supporting Information. The code of the simulation study is available from the corresponding author upon reasonable request.

## Endnotes

^1^
Since exponential BSSR simulations have been considered in previous simulation studies, we report our exponential simulation results in Appendix A.4. To summarize, as anticipated all BSSR methods were unbiased in the exponential setting. However, the variability in the estimated number of events at trial end increased with model complexity.
