# Supplementary material for: Flexible Spline Models for Blinded Sample Size Reestimation in Event‐Driven Clinical Trials
Source: Pharm Stat. 2024 Dec 11;24(2):e2459. doi: 10.1002/pst.2459 (PMC11893375; doi:10.1002/pst.2459)
Supplement: Supplementary file 1 — Data S1 Supporting Information. [file PST-24-0-s002.pdf]

A Appendix

A.1 Details of the simulation set up

Assumptions for the data generation We assumed that a total of 1530 patients would be recruited over a recruitment period of 20 months with a 2:1 treatment allocation. The planned length of the study for this event-driven design was 39 months. We generated data under the assumed alternative scenario of $\theta = 0.7$ and the null hypothesis scenario of $\theta = 1$. Proportional hazards were assumed for the data generation. We assumed that a log-rank test is used for the primary analysis. Based on the Schoenfeldt formula 374 events are necessary to obtain a power of 90% for a two-sided test at 5% significance level given a 2:1 randomization.

Design options In more detail the design options were as follows:

1. Flexible BSSR: Time-to-event and time-to-censoring are modeled based on independent Royston-Parmar spline models. Pooled parameter estimates of the survival function are used (no splitting).

2. Weibull BSSR: Time-to-event is modeled based on a Weibull model and time-to-censoring is modeled based on an exponential model. Group-specific parameter estimates of the survival function are used (splitting).

3. Exponential BSSR: Time-to-event and time-to-censoring are modeled based on independent exponential models. Group-specific parameter estimates of the survival function are used (splitting).

4. Fixed sample size design: No BSSR is carried out. The study is carried out using the sample size of $n = 1530$ calculated based on the planning assumption.

The Exponential BSSR and Weibull BSSR served as a comparator to the proposed flexible BSSR. A fixed sample size design was included to assess the consequences of not carrying out any sample size review in a setting with incorrect planning assumptions. For all BSSR methods the maximum number of additional recruitment months was set to 6 months, which corresponds to a maximum of 612 additional patients. Additional recruitment was considered in discrete steps of 1 month with 102 patients being recruited each month.

Software All simulations were carried out in R 4.0.2. (R Core Team, 2017). Survival data were generated using the 'simsurv' package (Brilleman et al., 2021). Exponential and Weibull models were fit using the 'survival' package (Therneau, 2020). Spline models were fit using the 'flexsurv' package (Jackson, 2016). Numerical integration was performed using the built-in R function *integrate()*, which performs adaptive quadrature (R Core Team, 2017).

A.2 Parameter sets for data-generation in the simulation study

In all simulation scenarios the censoring times were generated from an exponential distribution with 20% censoring at 24 months. The rate parameter of the corresponding exponential distribution was 0.0093.

As for the survival distribution, the probability of experiencing an event ranged from 20%, 21%, ..., to 30% at 24 months. The corresponding parameter sets for the different simulation scenarios (exponential, Weibull, Gompertz) are presented in the tables below.

Table A1: Parameter set for the data generation in the simulation scenarios with exponentially distributed survival times, where $S(t) = e^{-\lambda t}$.

Exponential Survival	
Events	λ
20%	0.0093
21%	0.0098
22%	0.0104
23%	0.0109
24%	0.0114
25%	0.0120
26%	0.0125
27%	0.0131
28%	0.0137
29%	0.0143
30%	0.0149

Table A2: Parameter set for the data generation in the simulation scenarios with Weibull distributed survival times, where $S(t) = exp\{-\lambda t^{\gamma}\}$. γ is is the shape parameter of the Weibull distribution. When $\gamma = 1$ the model reduces to an exponential model. If $\gamma > 1$ the hazard increases monotonically and if $\gamma < 1$ it decreases monotonically (Collett, 2015, Ch. 5).

Weibull Survival				
	Increasing Hazards		Decreasing Hazards	
Events	λ	γ	λ	γ
20%	0.0093	1.0000	0.0149	0.8524
21%		1.0173		0.8697
22%		1.0338		0.8862
23%		1.0497		0.9022
24%		1.0651		0.9175
25%		1.0799		0.9324
26%		1.0943		0.9467
27%		1.1082		0.9606
28%		1.1217		0.9741
29%		1.1348		0.9872
30%		1.1476		1.0000

Table A3: Parameter set for the data generation in the simulation scenarios with Gompertz distributed survival times, where $S(t) = exp\{\frac{\lambda}{\eta}(1-e^{\eta t})\}$. η is is the shape parameter of the Gompertz distribution. When $\eta = 0$ the model reduces to an exponential model. If $\eta > 0$ the hazard increases monotonically and if $\eta < 0$ it decreases monotonically (Collett, 2015, Ch. 5).

Gompertz Survival				
	Increasing Hazards		Decreasing Hazards	
Events	λ	η	λ	η
20%	0.0093	0.0000	0.0149	-0.0427
21%		0.0045		-0.0373
22%		0.0088		-0.0322
23%		0.0128		-0.0274
24%		0.0167		-0.0229
25%		0.0203		-0.0186
26%		0.0238		-0.0145
27%		0.0272		-0.0107
28%		0.0304		-0.0070
29%		0.0335		-0.0034
30%		0.0364		0.0000

A.3 Type I error rates

We found no indication of an increased type I error rate in any of the simulation scenarios considered.

Table A4: Simulated type I error rates summarized across the 11 exponential simulation scenarios with event probabilities from 20% to 30% (10,000 simulations per scenario). The nominal significance level is 0.05 (two-sided). SD is the standard deviation of the rejection probabilities across the 11 scenarios.

Method	Mean	SD	Minimum	Maximum
Fixed Design	0.0499	0.0028	0.0452	0.0537
Exponential BSSR	0.0505	0.0028	0.0455	0.0536
Weibull BSSR	0.0504	0.0026	0.0451	0.0535
1-Knot Spline BSSR	0.0501	0.0024	0.0463	0.0540
2-Knot Spline BSSR	0.0500	0.0023	0.0459	0.0536
3-Knot Spline BSSR	0.0502	0.0023	0.0471	0.0530

Table A5: Simulated type I error rates summarized across the 11 decreasing hazards Weibull simulation scenarios with event probabilities from 20% to 30% (10,000 simulations per scenario). The nominal significance level is 0.05 (two-sided). SD is the standard deviation of the rejection probabilities across the 11 scenarios.

Method	Mean	SD	Minimum	Maximum
Fixed Design	0.0507	0.0017	0.0475	0.0528
Exponential BSSR	0.0508	0.0019	0.0469	0.0528
Weibull BSSR	0.0510	0.0015	0.0482	0.0529
1-Knot Spline BSSR	0.0504	0.0016	0.0478	0.0527
2-Knot Spline BSSR	0.0508	0.0012	0.0484	0.0525
3-Knot Spline BSSR	0.0505	0.0018	0.0471	0.0526

Table A6: Simulated type I error rates summarized across the 11 increasing hazards Weibull simulation scenarios with event probabilities from 20% to 30% (10,000 simulations per scenario). The nominal significance level is 0.05 (two-sided). SD is the standard deviation of the rejection probabilities across the 11 scenarios.

Method	Mean	SD	Minimum	Maximum
Fixed Design	0.0497	0.0018	0.0452	0.0517
Exponential BSSR	0.0500	0.0009	0.0485	0.0511
Weibull BSSR	0.0502	0.0011	0.0485	0.0511
1-Knot Spline BSSR	0.0502	0.0009	0.0491	0.0519
2-Knot Spline BSSR	0.0500	0.0015	0.0478	0.0528
3-Knot Spline BSSR	0.0497	0.0016	0.0475	0.0518

Table A7: Simulated type I error rates summarized across the 11 decreasing hazards Gompertz simulation scenarios with event probabilities from 20% to 30% (10,000 simulations per scenario). The nominal significance level is 0.05 (two-sided). SD is the standard deviation of the rejection probabilities across the 11 scenarios.

Method	Mean	SD	Minimum	Maximum
Fixed Design	0.0507	0.0024	0.0473	0.0550
Exponential BSSR	0.0506	0.0022	0.0475	0.0549
Weibull BSSR	0.0502	0.0024	0.0465	0.0548
1-Knot Spline BSSR	0.0497	0.0021	0.0465	0.0543
2-Knot Spline BSSR	0.0501	0.0022	0.0468	0.0537
3-Knot Spline BSSR	0.0502	0.0016	0.0473	0.0533

Table A8: Simulated type I error rates summarized across the 11 increasing hazards Gompertz simulation scenarios with event probabilities from 20% to 30% (10,000 simulations per scenario). The nominal significance level is 0.05 (two-sided). SD is the standard deviation of the rejection probabilities across the 11 scenarios.

Method	Mean	SD	Minimum	Maximum
Fixed Design	0.0497	0.0024	0.0452	0.0531
Exponential BSSR	0.0501	0.0021	0.0469	0.0535
Weibull BSSR	0.0498	0.0016	0.0471	0.0528
1-Knot Spline BSSR	0.0501	0.0022	0.0465	0.0536
2-Knot Spline BSSR	0.0498	0.0022	0.0471	0.0536
3-Knot Spline BSSR	0.0498	0.0024	0.0462	0.0547

A.4 Exponential simulation results

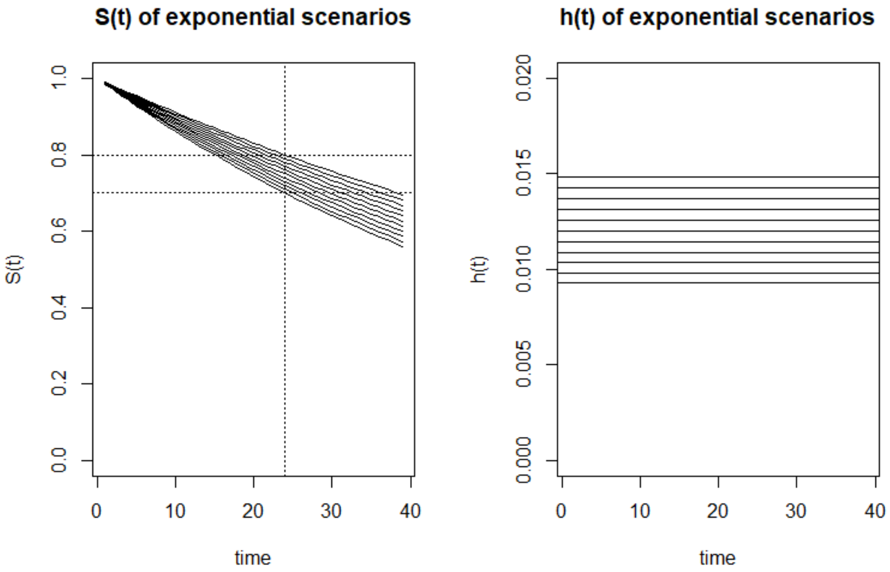

Figure A.1: Survival and hazard functions of the simulated exponential scenarios. The vertical dotted line indicates the reference month 24, at which the probability of experiencing the event is between 20% and 30% (horizontal dotted lines).

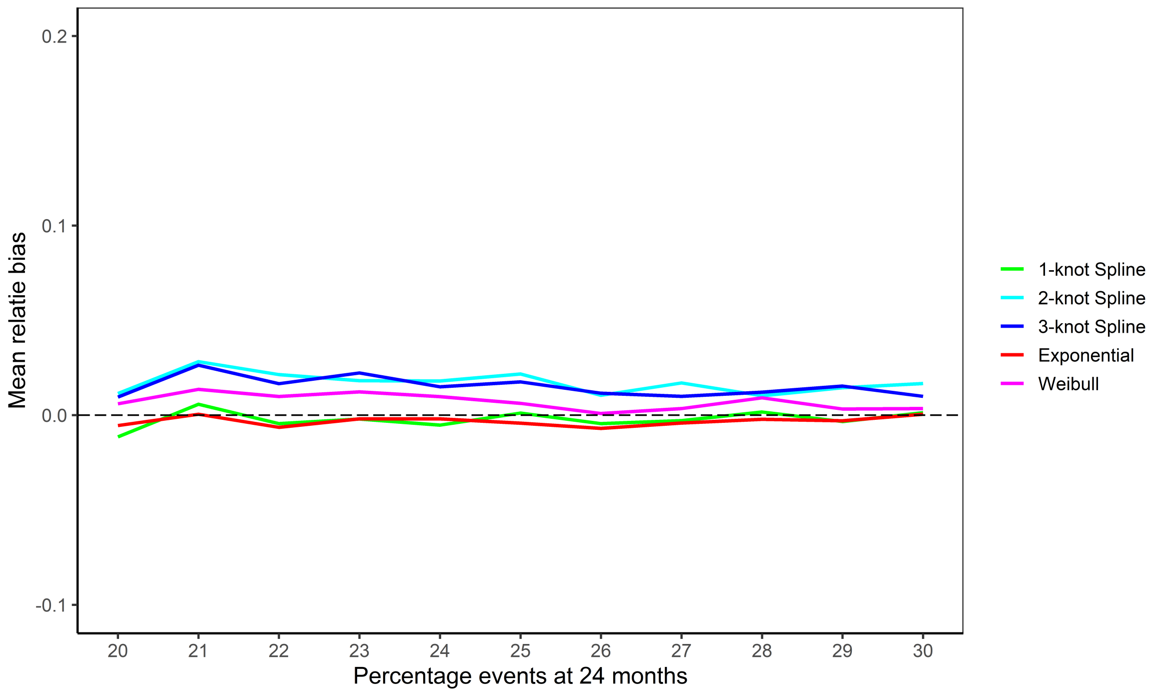

Figure A.2: Mean relative bias in the estimated number of events at trial end in the simulated exponential scenarios based on the BSSR models (1,000 replications per scenario). Splines models were fit with 1, 2 and 3 knots (denoted by Splines1, Splines2 and Splines3, respectively). Note that no sample size reestimation was carried out in the fixed design. The expected number of events in the simulation scenarios ranged from 246.8 (20% events) to 372.3 (30% events).

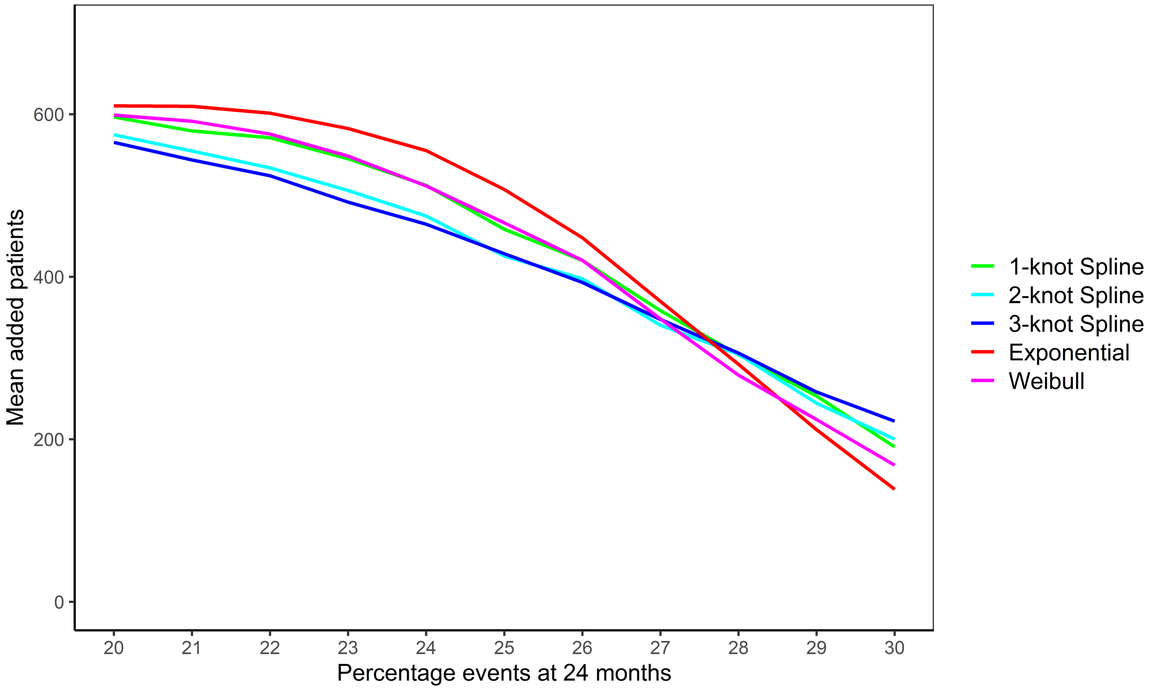

Figure A.3: Mean number of additional patients in the exponential scenarios added by the different BSSR models (1,000 replications per scenario). The maximum number of patients that could be added was 612. Note that no patients were added in the fixed design.

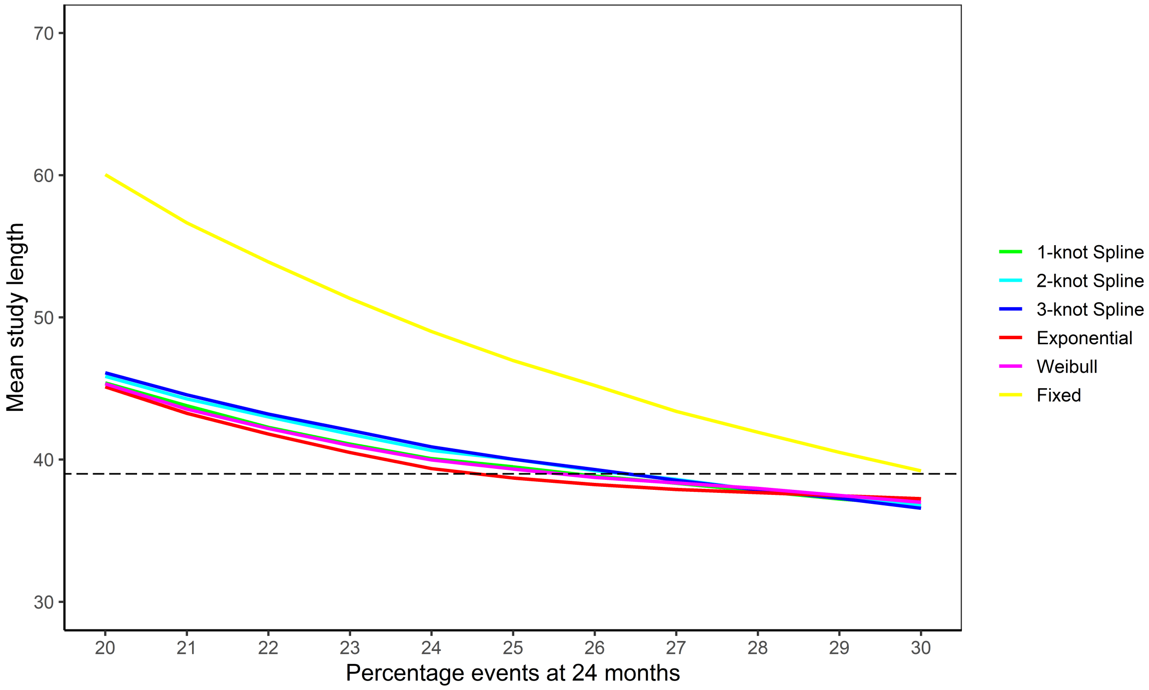

Figure A.4: Mean study lengths in the exponential scenarios based on the BSSR models and the fixed design (1,000 replications per scenario). The trial finished once 374 events were observed and the goal was to finish in 39 months (black dotted line).

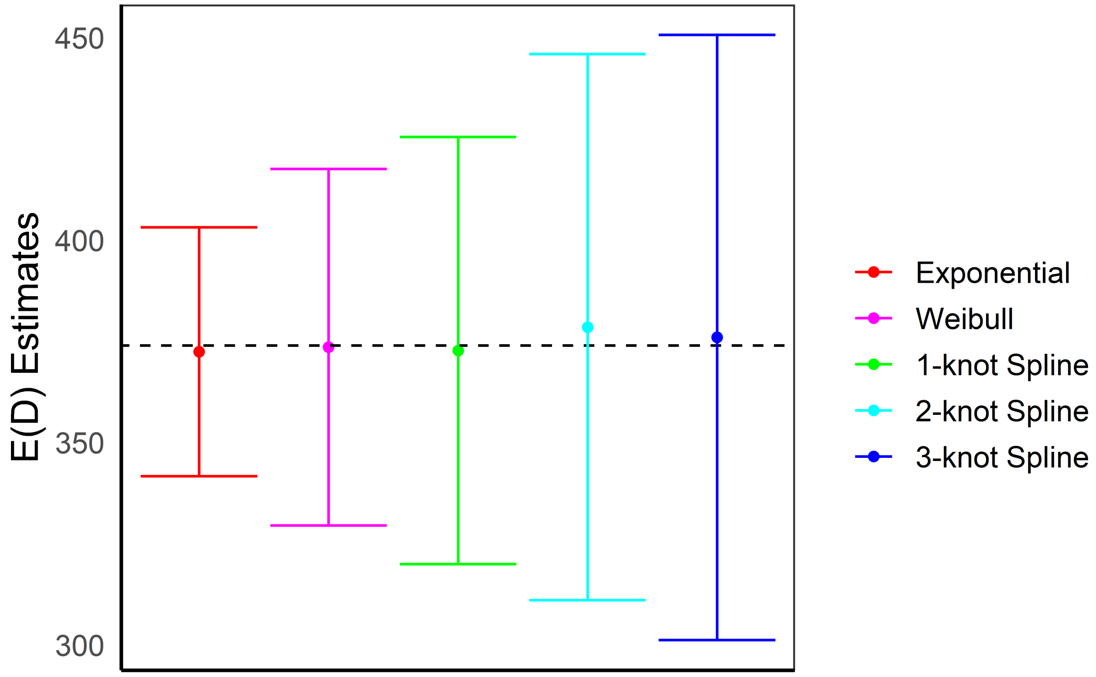

Figure A.5: Mean and standard deviation (depicted as error bars) of the estimated number of events based on the different BSSR models across 1000 simulation runs in the exponential setting with an event percentage of 30% at 24 months in the control group (corresponding to about 374 expected events after 39 months, see dotted line). All methods were unbiased, but the variability in the estimated number of events increases with model complexity.

A.5 Weibull simulation results

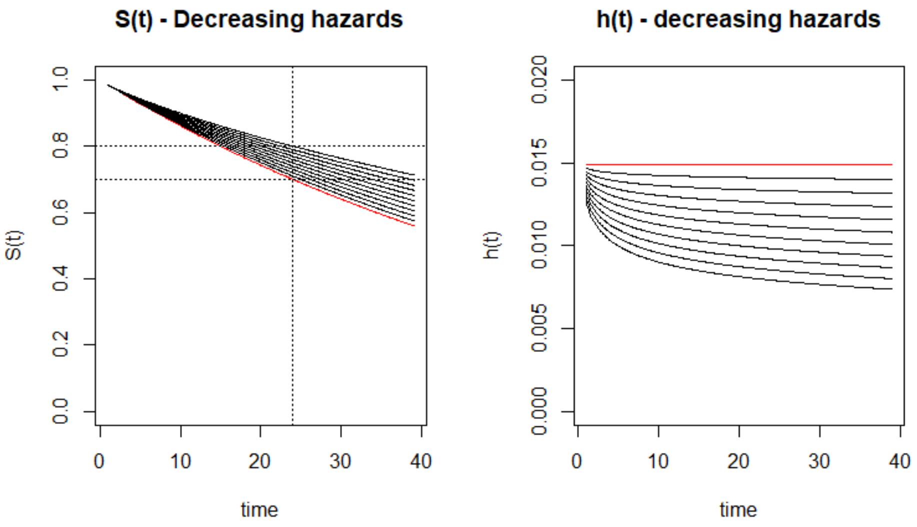

Figure A.6: Survival and hazard functions of the simulated Weibull scenarios with decreasing hazards. The red line indicates the exponential distribution with 30% events at 24 months, which is chosen as reference point (Weibull shape parameter equal to 1). From there the Weibull shape parameter is gradually decreased until there are only 20% events at 24 months. The resulting decreasing hazards are shown in the right panel.

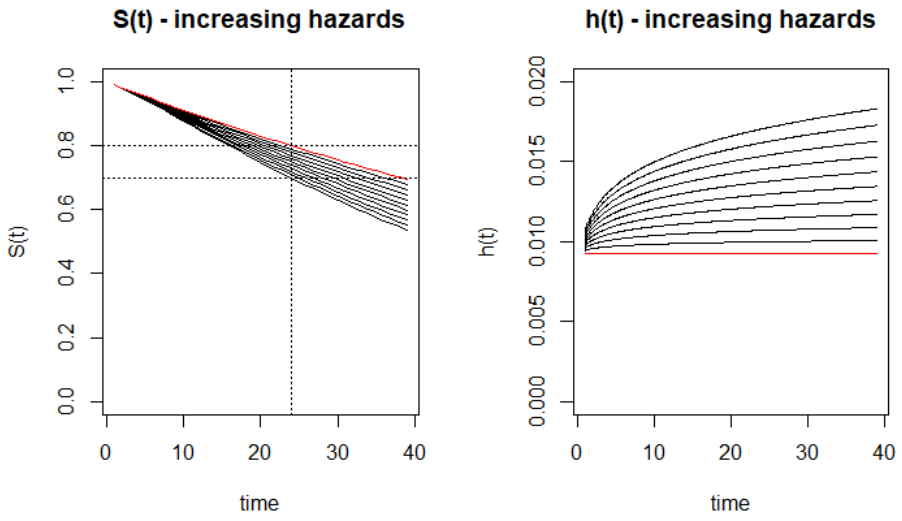

Figure A.7: Survival and hazard functions of the simulated Weibull scenarios with increasing hazards. The red line indicates the exponential distribution with 20% events at 24 months, which is chosen as reference point (Weibull shape parameter equal to 1). From there the Weibull shape parameter is gradually increased until there are 30% events at 24 months. The resulting increasing hazards are shown in the right panel.

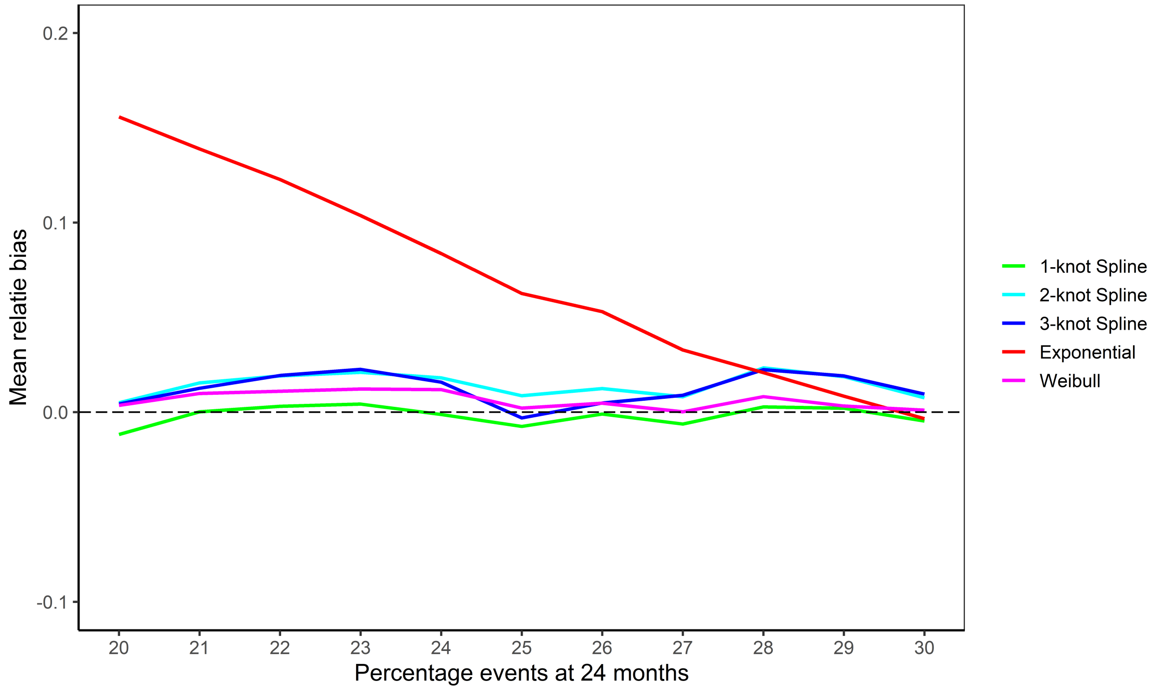

Figure A.8: Mean relative bias in the estimated number of events at trial end in the simulated decreasing hazards Weibull scenarios based on the BSSR models (1,000 replications per scenario). The Royston-Parmar spline model was fit with 1, 2 and 3 knots. Note that no sample size reestimation was carried out in the fixed design. The expected number of events in the simulation scenarios ranged from 245.1 (20% events) to 372.3 (30% events).

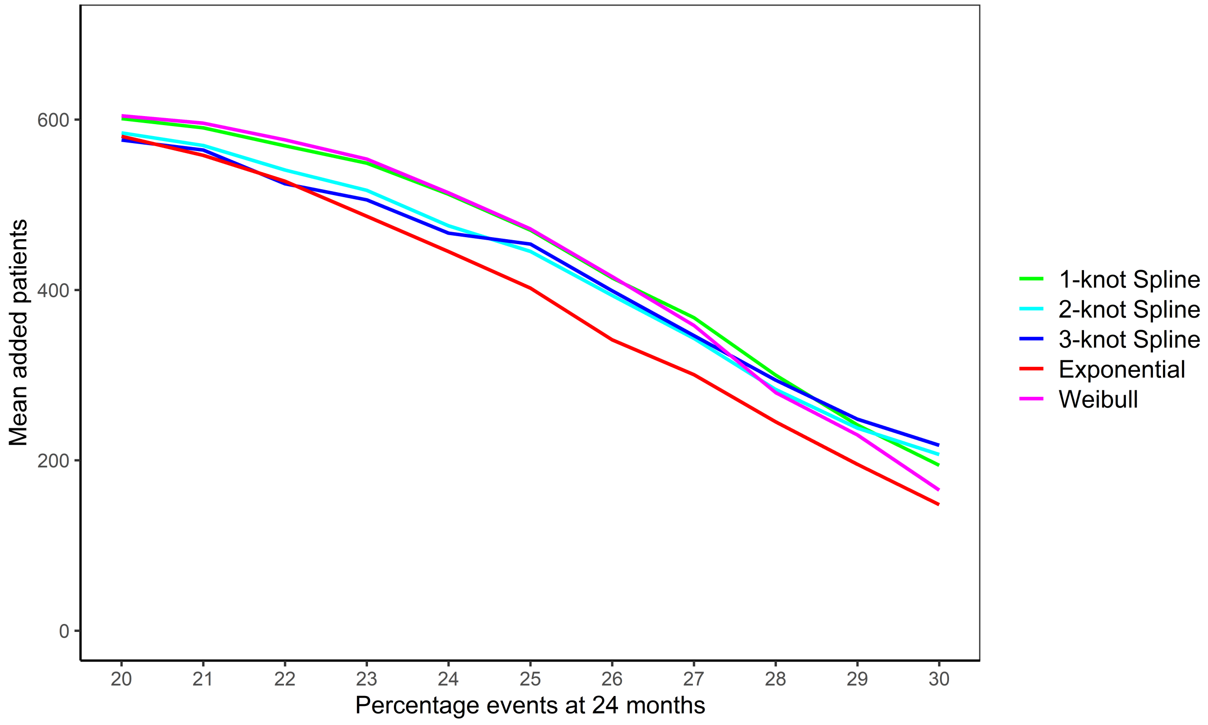

Figure A.9: Mean number of additional patients in the Weibull scenarios with decreasing hazards added by the different BSSR models (1,000 replications per scenario). The maximum number of patients that could be added was 612. Note that no patients were added in the fixed design.

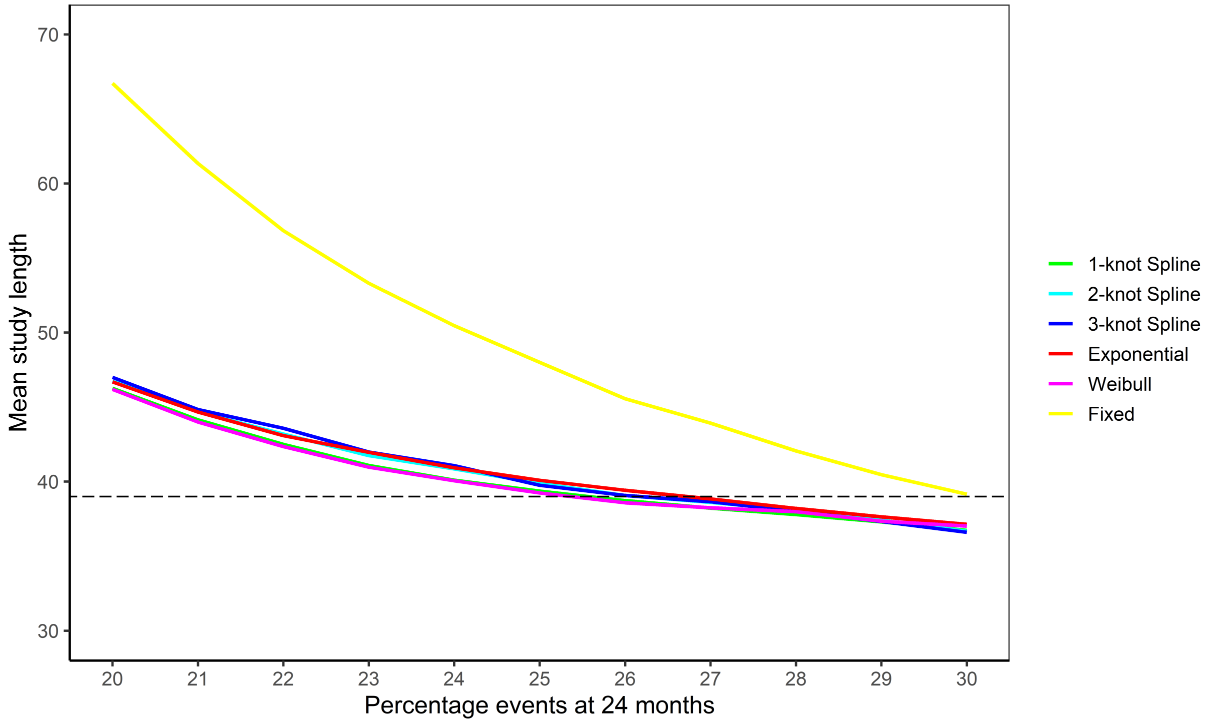

Figure A.10: Mean study lengths in the Weibull scenarios with decreasing hazards based on the BSSR models and the fixed design (1,000 replications per scenario). The trial finished once 374 events were observed and the goal was to finish in 39 months (black dotted line).

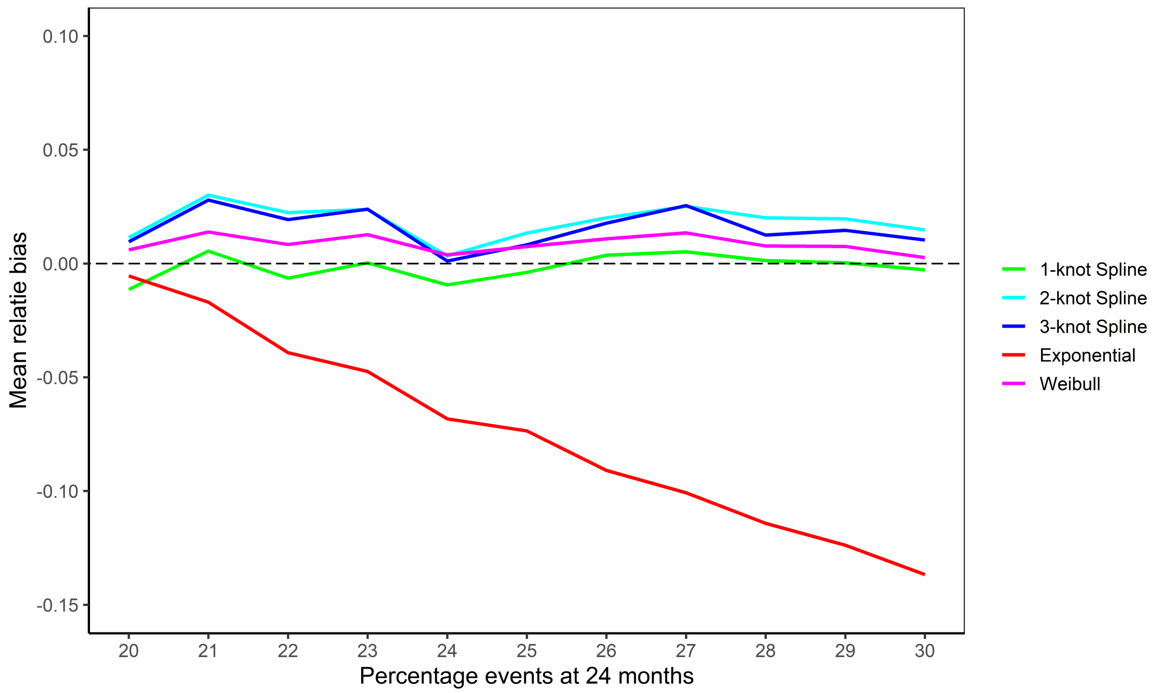

Figure A.11: Mean relative bias in the estimated number of events at trial end in the simulated increasing hazards Weibull scenarios based on the BSSR models (1,000 replications per scenario). The Royston-Parmar spline model was fit with 1, 2 and 3 knots. Note that no sample size reestimation was carried out in the fixed design. The expected number of events in the simulation scenarios ranged from 246.8 (20% events) to 374.9 (30% events).

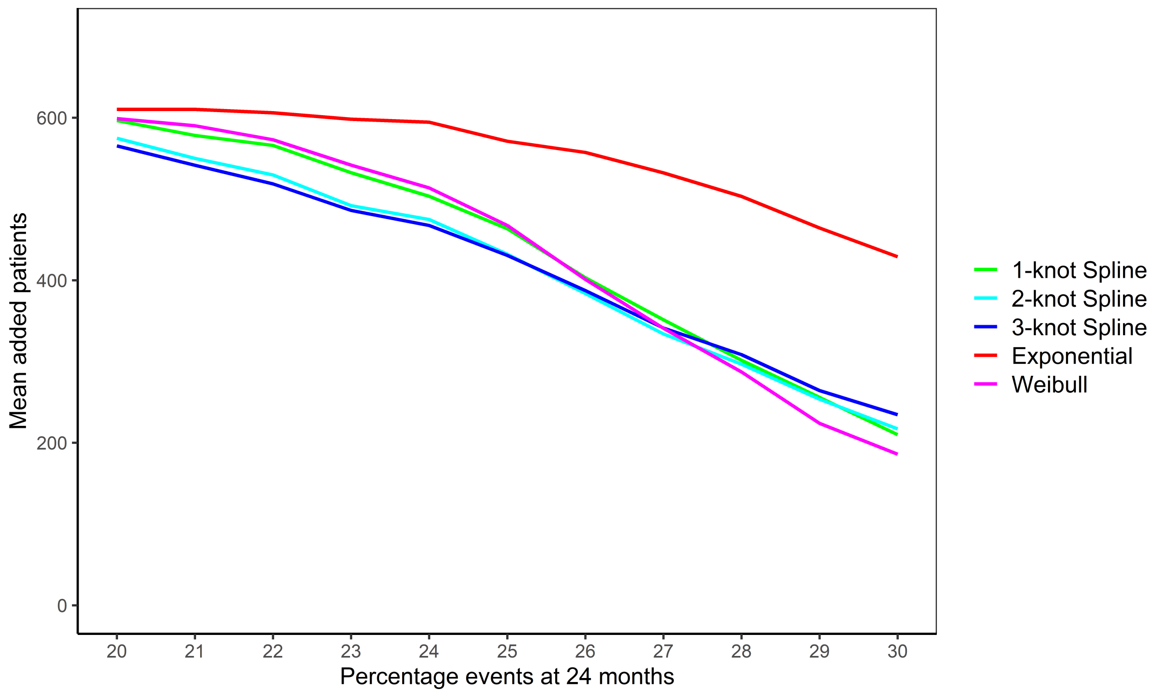

Figure A.12: Mean number of additional patients in the Weibull scenarios with increasing hazards added by the different BSSR models (1,000 replications per scenario). The maximum number of patients that could be added was 612. Note that no patients were added in the fixed design.

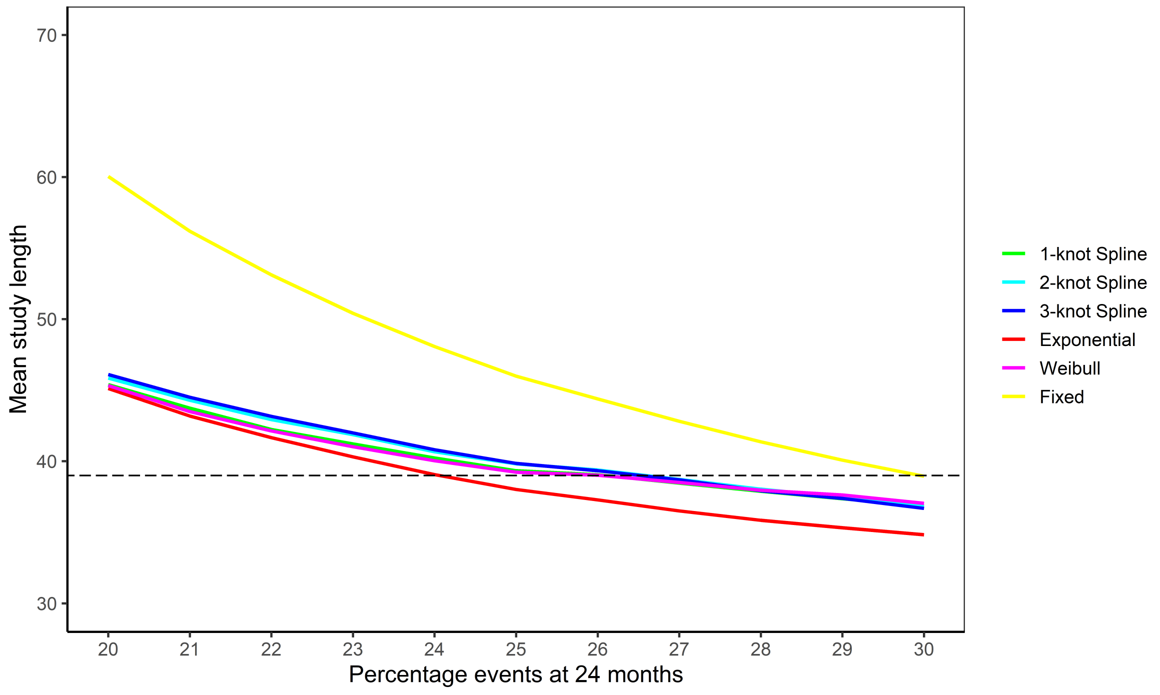

Figure A.13: Mean study lengths in the Weibull scenarios with increasing hazards based on the BSSR models and the fixed design (1,000 replications per scenario). The trial finished once 374 events were observed. The trial finished once 374 events were observed and the goal was to finish in 39 months (black dotted line).

A.6 Gompertz simulation results

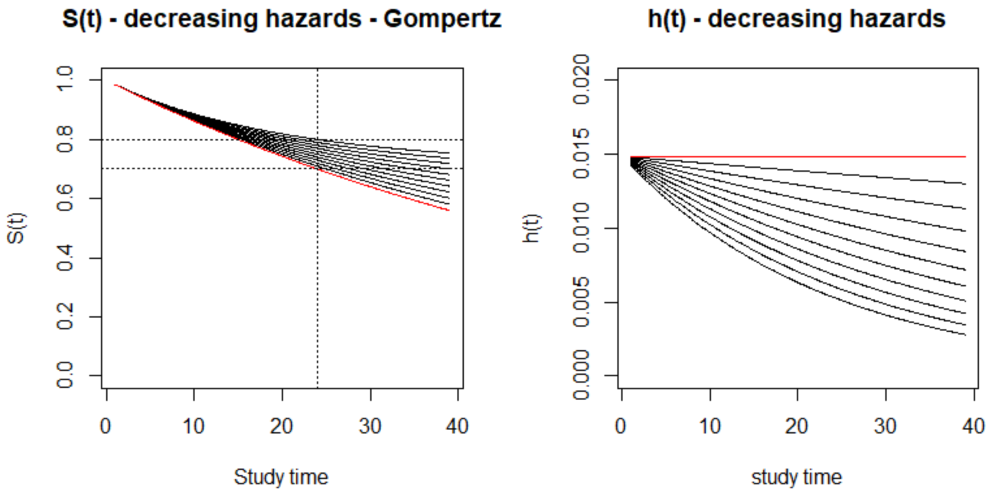

Figure A.14: Survival and hazard functions of the simulated Gompertz scenarios with decreasing hazards. The red line indicates the exponential distribution with 30% events at 24 months, which is chosen as reference point (Gompertz shape parameter equal to 0). From there the Gompertz shape parameter is gradually decreased until there is only 20% events at 24 months. The resulting decreasing hazards are shown in the right panel.

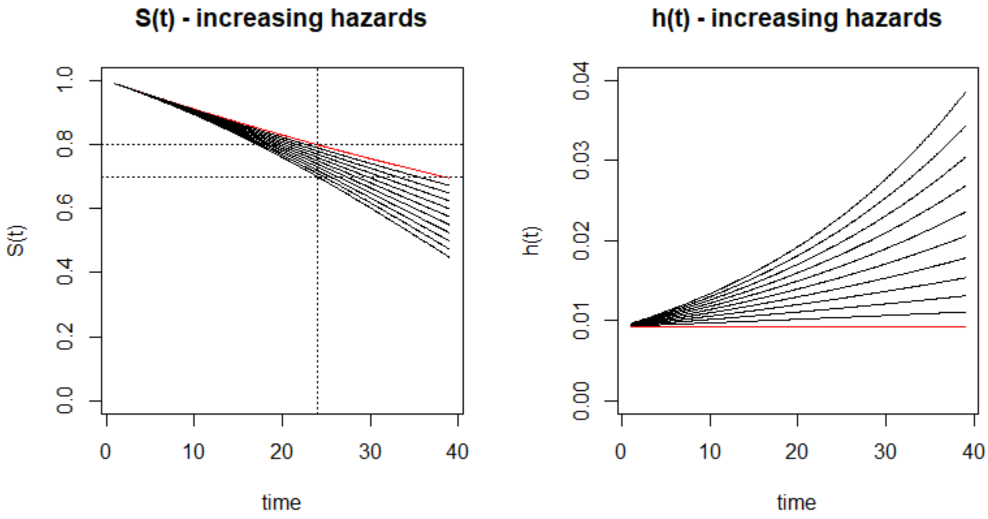

Figure A.15: Survival and hazard functions of the simulated Gompertz scenarios with increasing hazards. The red line indicates the exponential distribution with 20% events at 24 months, which is chosen as reference point (Gompertz shape parameter equal to 1). From there the Gompertz shape parameter is gradually increased until there is 30% events at 24 months. The resulting increasing hazards are shown in the right panel.

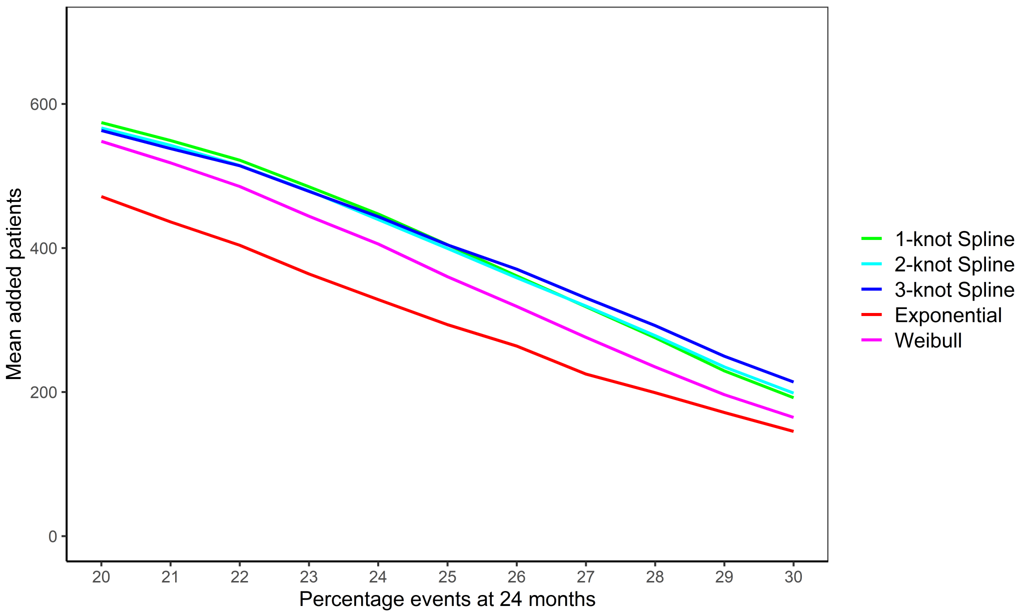

Figure A.16: Mean number of additional patients in the Gompertz scenarios with decreasing hazards added by the different BSSR models (10,000 replications per scenario). The maximum number of patients that could be added was 612. Note that no patients were added in the fixed design.

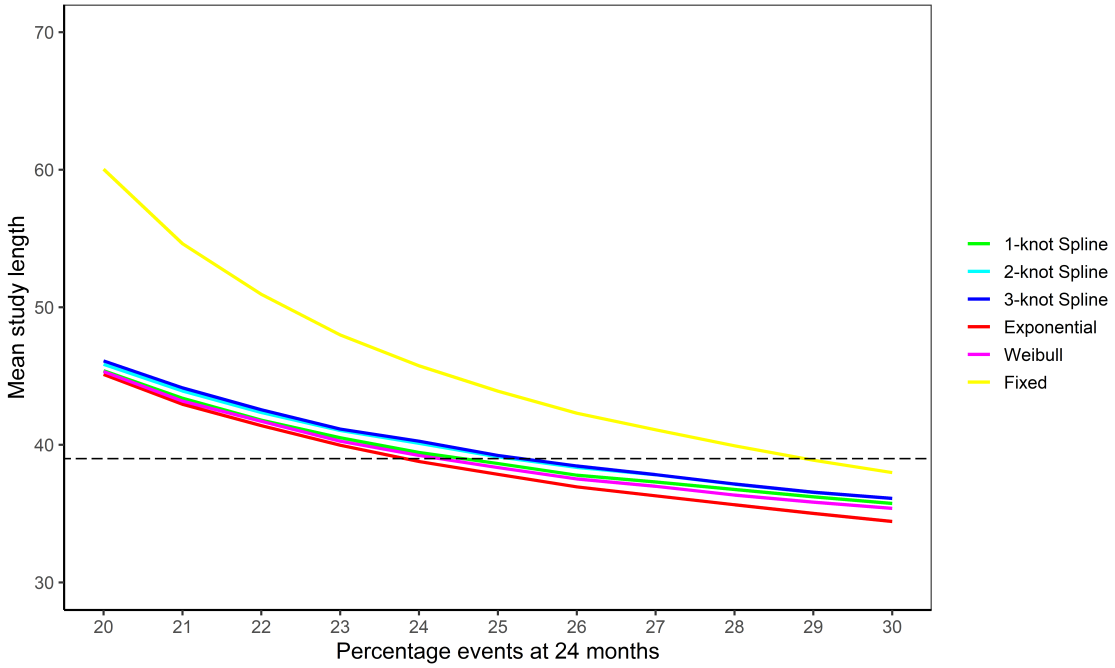

Figure A.17: Mean study lengths in the Gompertz scenarios with increasing hazards based on the BSSR models and the fixed design (10,000 replications per scenario). The trial finished once 374 events were observed. The trial finished once 374 events were observed and the goal was to finish in 39 months (black dotted line).

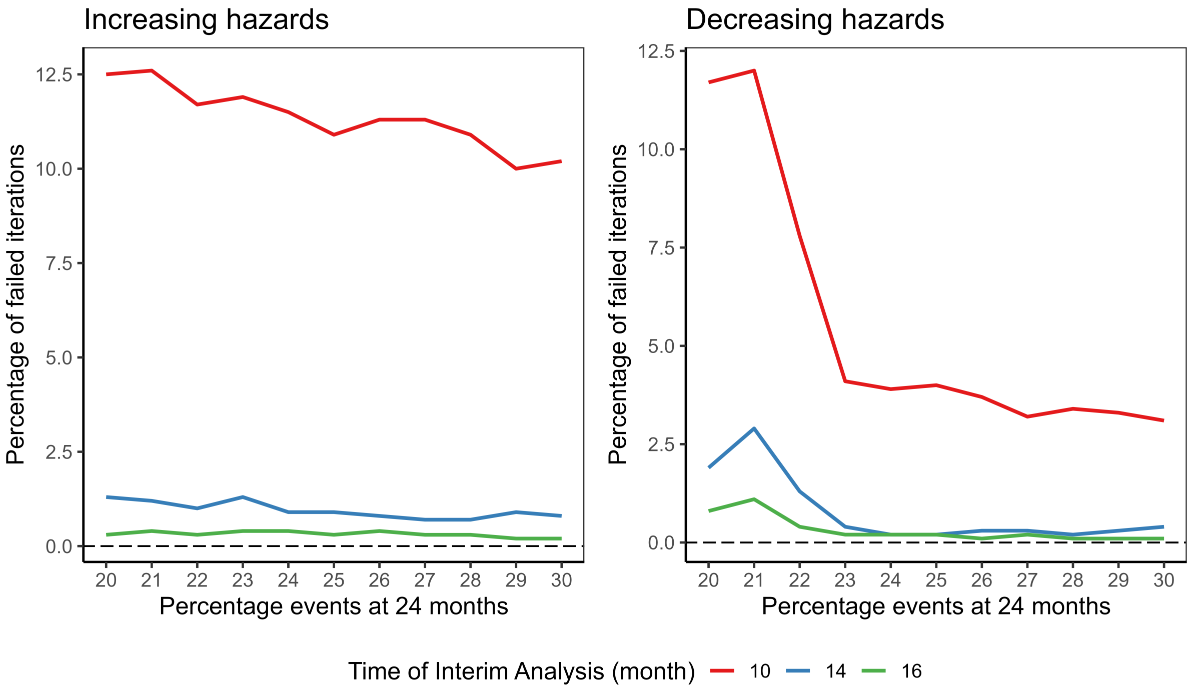

Figure A.18: Sensitivity analyses for the Gompertz simulation, with interim analyses conducted at earlier time points (16, 14, and 10 months), resulting in smaller sample sizes (1110, 903, and 495 patients, respectively). The figure shows the percentage of simulation iterations in which the flexible spline model with up to three internal knots could not be estimated (10,000 replications per scenario).

A.7 Case Study

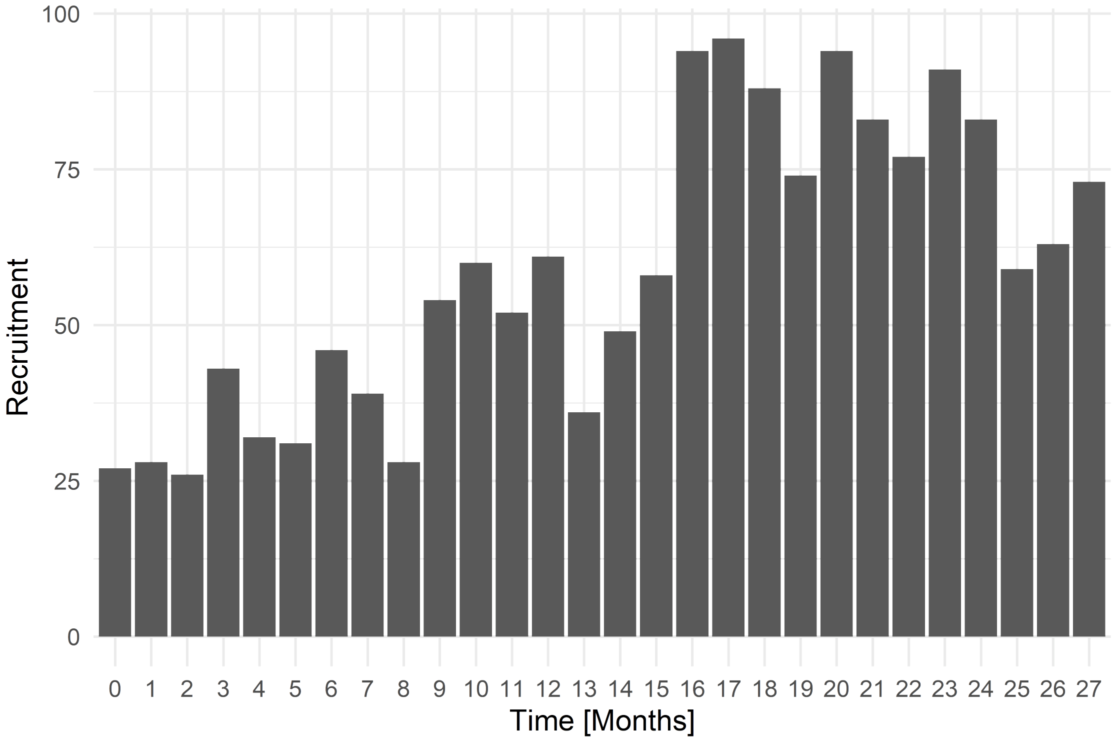

Figure A.19: Simulated monthly enrollment numbers for the 28 months enrollment period that was reported in the SPMS study of Kappos et al. (2018).

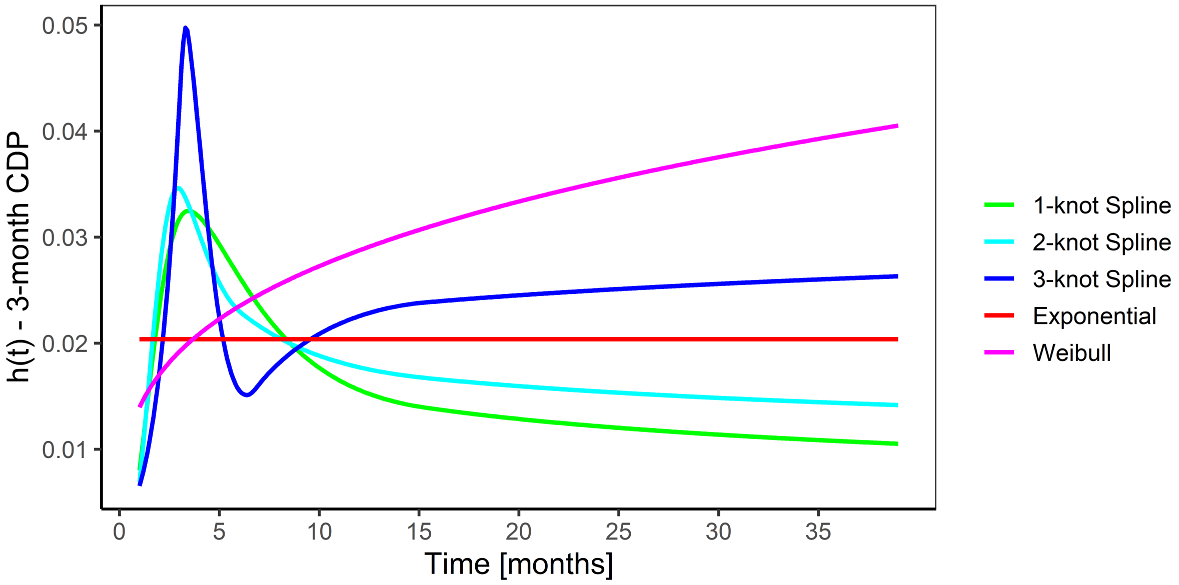

Figure A.20: Estimated hazard function based on the interim data of the secondary progressive multiple sclerosis (SPMS) data based on standard parametric models (Exponential, Weibull) as well as the Royston-Parmar spline model with 1, 2 or 3 knots.

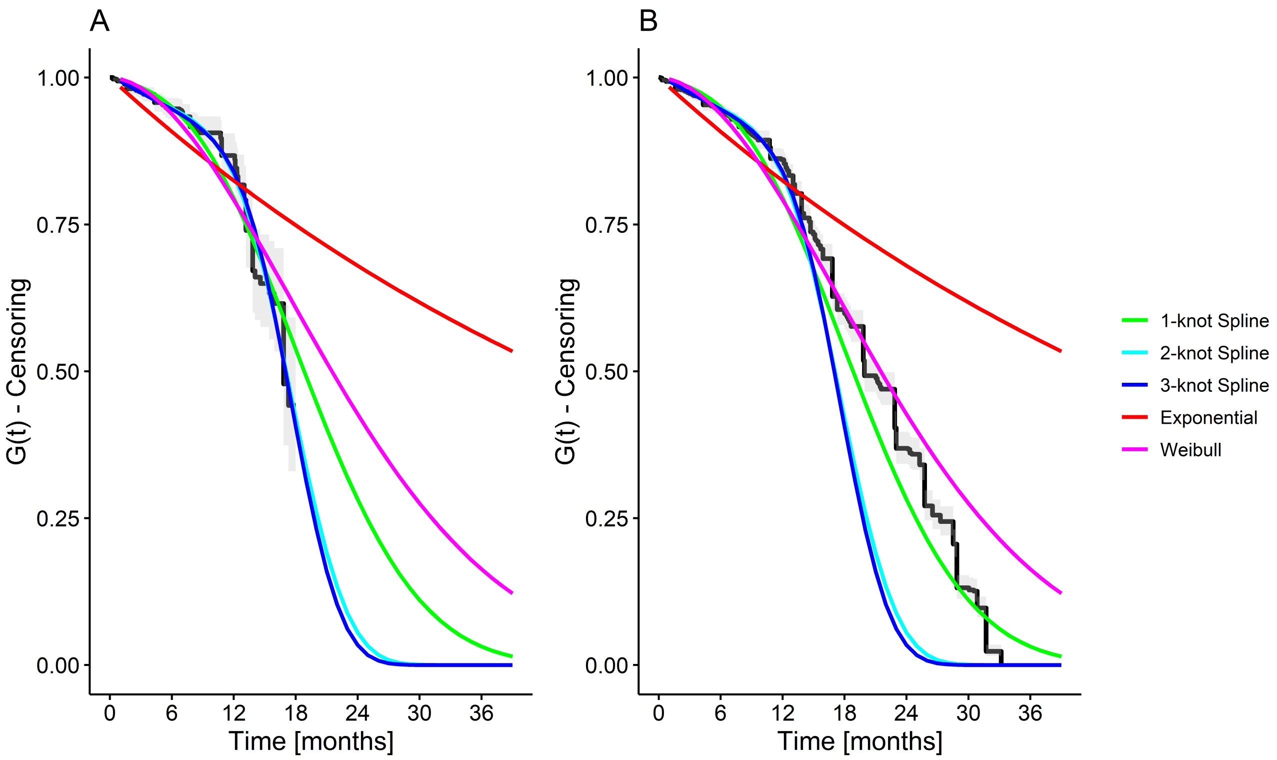

Figure A.21: Censoring modeling of the interim data of the secondary progressive multiple sclerosis (SPMS) data based on standard parametric models (Exponential, Weibull) as well as the Royston-Parmar spline model with 1, 2 or 3 knots. The predicted values based on the different models are shown in comparison to the Kaplan-Meier curve of A) the interim data, and B) the full dataset.

A.8 Literature review: Simulation and real-world studies evaluating the Royston-Parmar spline model

Table A9: Summary of simulation and real-world studies assessing the (extrapolation) performance of the Royston-Parmar spline model in datasets with different sample sizes and different lengths of follow-up available for model estimation.

	**Rutherford et al. (2015)**	**Rutherford et al. (2020)**	**Kearns et al. (2021)**	**Chen et al. (2024)**
**Setting**	Theoretical setting	Economic evaluation setting	Economic evaluation setting	Economic evaluation setting (oncology)
**Data**	Simulated data from<br>A) Weibull distributions (simple hazard shapes) and<br>B) Mixture Weibull distributions (complex hazard shapes)	Simulated data from<br>A) Weibull distributions (simple hazard shapes) and<br>B) Mixture Weibull distributions (complex hazard shapes)<br><br>Further varied:<br>1) Survival rates (low / medium)<br>2) Heterogeneity (low / high)	Simulated data from a single Mixture Weibull distribution	Real-world cancer registry data; 15 different subcohorts based on cancer type and age range of participants.<br>Follow-up data were artificially censored to generate interim data. Extrapolation was compared to real, observed long-term outcomes.
**Sample size**	N = 300 / 3.000 / 30.000	N = 100 / 500	N = 100 / 300 / 600	N = 218 – 29.749 (depending on subcohort)
**Follow-up**	10 years	3 years	2 / 3 / 4 years	2 / 3 / 5 years
**Models**	Spline model (PH) with 0-9 knots	A) Spline model (PH) with 0-4 knots<br>B) Standard parametric models<br>C) Weibull cure model<br>D) Spline model with relative survival framework	A) Spline model (PH / PO) with 1-5 knots (selected automatically via AIC)<br>B) Standard parametric models<br>C) Fractional Polynomials<br>D) Generalised additive models<br>E) Dynamic survival models	A) Spline model (PH / PO / probit) with 4 internal knots<br>B) Standard parametric models<br><br>Models were additionally also fit in a relative survival framework (not considered here)
**Exrapolation**	None	Mean overall survival time (up to 80 years extrapolation)	10-20 years extrapolation	10 years extrapolation
**Metric**	Absolute area difference between true and fitted survival / hazard functions	Bias in estimated mean overall survival time	Mean squared error / bias in logarithm of log-transformed time-varying hazard	Difference in 10-year survival (extrapolated – observed)

**Results**	Even in smaller simulated clinical trials (N=300) there was minimal bias in the estimated hazard / survival functions. There was not a large difference in performance compared to when larger clinical trials (N=3.000 / 30.000) were simulated, given that a sufficient number of knots was used. However, occasional overfitting was more likely to occur with flexible spline models (> 3 or 4 knots) in smaller clinical trials.	Given a 3-year follow-up the spline models extrapolated the survival time well in Weibull simulation scenarios, also with the smaller sample sizes (N = 100 / 500). However, in a small clinical trial (N = 100) with a lower event rate overfitting was more likely for spline models with an increasing number of internal knots. When high heterogeneity was present or data were simulated from a cure model the spline model did not extrapolate well. In general, the spline model performed similar to a simple Weibull model in the settings considered here.	In the setting considered here the spline model was unable to capture the long-term increase in the hazard function and generally performed similarly to standard parametric models. Shorter available follow-up times (2 vs 4 years) did not drastically affect the spline model, though performance improved slightly with longer follow-up times available. A smaller sample size (n = 100) lead to larger variability, which was somewhat more pronounced for the spline models than for standard parametric models.	Considering all 15 subcohorts, the 4-knot PH spline model performed poorly when the available follow-up time was short (2 years). This was the same for a Weibull model, whereas a simple exponential model was more robust in settings with shorter follow-up times. With medium (3 years) follow-up, the exponential and spline model performed similarly, whereas a Weibull model still performed poorly. At longer follow-up (5 years) the spline model performed best.<br>In the chronic myeloid leukemia (CML) subcohorts with smaller sample sizes (N = 218 – 359) the 4-knot spline model performed decently, also at shorter follow-up times (2 years). However, so did a simple exponential model, even though it did not fit the observed hazard function well.
**Limitations**	-Long follow-up (10 years) was available<br>-Available follow-up duration was fixed<br>-No extrapolation was considered (only observed sample fit)	-Available follow-up duration was fixed<br>-Extrapolation was carried out with a very long time horizon (overall mean survival time), which is different from extrapolation in a BSSR setting	-Only a single data-generating mechanism was considered<br>-Extrapolation was carried out with a long time horizon (10-20 years)<br>-The number of knots and the scale of the spline model were selected automatically based on the AIC	-Only data from cancer patients was used here<br>-Except for the CML subcohort, the sample sizes were quite large (N = 1808 – 29.749)<br>-Per default, a very flexible 4-knot spline model was used

PH = Proportional hazards; PO = Proportional odds; BSSR = Blinded sample size review

References

Brilleman, S. L.,Wolfe, R., Moreno-Betancur, M., and Crowther, M. J. (2021). Simulating survival data using the simsurv r package. *Journal of Statistical Software*, 97, 1-27.

Chen, E. Y. T., Leontyeva, Y., Lin, C. N., Wang, J. D., Clements, M. S., & Dickman, P. W. (2024). Comparing Survival Extrapolation within All-Cause and Relative Survival Frameworks by Standard Parametric Models and Flexible Parametric Spline Models Using the Swedish Cancer Registry. *Medical Decision Making*, 44(3), 269-282.

Jackson, C. H. (2016). flexsurv: a platform for parametric survival modeling in R. *Journal of Statistical Software*, 70(8), 1-33.

Kearns, B., Stevenson, M. D., Triantafyllopoulos, K., & Manca, A. (2021). Comparing current and emerging practice models for the extrapolation of survival data: a simulation study and case-study. *BMC medical research methodology*, *21*, 1-11.

R Core Team (2017). *R: A Language and Environment for Statistical Computing*. R Foundation for Statistical Computing, Vienna, Austria.

Rutherford, M. J., Crowther, M. J., & Lambert, P. C. (2015). The use of restricted cubic splines to approximate complex hazard functions in the analysis of time-to-event data: a simulation study. *Journal of Statistical Computation and Simulation*, *85*(4), 777-793.

Rutherford, M. J., Lambert, P. C., Sweeting, M. J., Pennington, R., Crowther, M. J., & Abrams, K. R., & Latimer, N.R. (2020). NICE DSU technical support document 21. Flexible methods for survival analysis. Retrieved from http://www.nicedsu.org.uk.

Therneau, T. M. (2020). *A Package for Survival Analysis in R*. R package version 3.2.7, Retrieved from https://CRAN.R-project.org/package=survival.
